# *N*-aldehyde-modified phosphatidylethanolamines generated by lipid peroxidation are robust substrates of *N*-acyl phosphatidylethanolamine phospholipase D

**DOI:** 10.1016/j.jlr.2025.100831

**Published:** 2025-05-21

**Authors:** Reza Fadaei, Annie C. Bernstein, Andrew N. Jenkins, Allison G. Pickens, Jonah E. Zarrow, Abdul-Musawwir Alli-Oluwafuyi, Keri A. Tallman, Sean S. Davies

**Affiliations:** 1 Department of Pharmacology, Vanderbilt University, Nashville, TN, USA; 2 College of Arts and Sciences, Vanderbilt University, Nashville, TN, USA; 3 Department of Cell Biology and Physiology, Brigham Young University, Provo, UT, USA; 4 Department of Plant and Wildlife Sciences, Brigham Young University, Provo, UT, USA; 5 Department of Chemistry, Vanderbilt University, Nashville, TN, USA; 6 Vanderbilt Institute of Chemical Biology, Vanderbilt University, Nashville, TN, USA

**Keywords:** reactive lipid aldehydes, lipid peroxidation, phospholipases, phosphatidylethanolamine, *N*-acyl-ethanolamines, oxidized phospholipids

## Abstract

N-acyl phosphatidylethanolamine-hydrolyzing phospholipase D (NAPE-PLD) hydrolyzes phosphatidylethanolamines (PEs) where the headgroup nitrogen has been enzymatically modified with acyl chains of four carbons or longer (*N*-acyl-PEs or NAPEs). The nitrogen headgroup of PE can also be nonenzymatically modified by reactive lipid aldehydes, thus forming *N*-aldehyde-modified PEs (NALPEs). Some NALPEs such as *N*-carboxyacyl-PEs are linked to PE via amide bonds similar to NAPEs, but others are linked by imine, pyrrole, or lactam moieties. Whether NAPE-PLD can hydrolyze NALPEs was unknown. We therefore characterized the major NALPE species formed during lipid peroxidation of arachidonic acid and linoleic acid and generated various NALPEs for characterization of their sensitivity to NAPE-PLD hydrolysis by reacting synthesized aldehydes with PE. We found that NAPE-PLD could act on NALPEs of various lengths and linkage types including those derived from PE modified by *N*-malondialdehyde, *N*-4-hydroxynonenal, *N*-4-oxo-nonenal, *N*-9-keto-12-oxo-dodecenoic acid, and *N*-15-E_2_-isolevuglandin. To assess the relative preference of NAPE-PLD for various NALPEs versus its canonical NAPE substrates, we generated a substrate mixture containing roughly equimolar concentrations of seven NALPEs as well as two NAPEs (*N*-palmitoyl-PE and *N*-linoleoyl-PE) and measured their rate of hydrolysis. Several NALPE species, including the *N*-4-hydroxynonenal-PE pyrrole species, were hydrolyzed at a similar rate as *N*-linoleoyl-PE, and many of the other NALPEs showed intermediate rates of hydrolysis. These results significantly expand the substrate repertoire of NAPE-PLD and suggest that it may play an important role in clearing products of lipid peroxidation in addition to its established role in the biosynthesis of *N*-acyl-ethanolamines.

Lipid peroxidation of polyunsaturated fatty acids (PUFAs) including linoleic acid and arachidonic acid generates a variety of lipid aldehydes including acetaldehyde, malondialdehyde (MDA), hexanal, 9-oxo-nonanoic acid (ONA), 4-oxo-2-nonenal (ONE), 4-hydroxynonenal (HNE), 9-hydroxy-12-oxo-dodecenoic acid (HODA), 9-keto-12-oxo-dodecenoic acid (KODA), and isolevuglandin (IsoLG) (1, 2, 3, 4, 5, 6, 7). Several of these aldehydes have been previously shown to react with primary amines including phosphatidylethanolamines (PEs) to form stable *N*-aldehyde-modified PE (NALPE) adducts, including MDA, HNE, and IsoLG (8, 9, 10). Peroxidation of arachidonic acid in the presence of PE or of HDL was also shown to generate a series of *N*-carboxyacyl-PEs (11, 12). While the precise mechanism for the formation of *N*-carboxyacyl-PEs has not been fully elucidated, the most likely mechanism is by the reaction of PE with aldehyde species that retain the carboxylate moiety of the original PUFA during their formation by lipid peroxidation and where the initial reaction with PE forms an imine (Schiff base [SB]) bond that is subsequently oxidized to an amide bond by peroxides (11, 12).

Several previously characterized NALPEs have proinflammatory and cytotoxic effects (11). Understanding how these NALPEs are degraded could lead to a better understanding of how inflammation is regulated. *N*-acyl PE-hydrolyzing phospholipase D (NAPE-PLD) is a zinc metallo-β-lactamase that catalyzes the hydrolysis of PEs that have been enzymatically modified on the headgroup nitrogen with acyl chains of four carbons or longer (NAPEs) (13, 14). NAPE-PLD’s substrate preferences contrast with those of phospholipase D1 and D2, which are serine hydrolase-type enzymes that act on unmodified PEs and phosphatidylcholines but not on NAPEs (14, 15, 16). Homologs of NAPE-PLD are found in yeast, worms, reptiles, birds, and mammals, indicating it is likely to have a highly conserved function in physiology (14, 17, 18, 19). We hypothesized that NAPE-PLD may be an important enzyme for degrading NALPEs based on three previous observations. First, that the putative substrate-binding site of NAPE-PLD is a large hydrophobic nook with several hydrophobic channels emanating from the active site that could potentially accommodate a wide variety of structures (20). Second, that fluorogenic probes used to monitor NAPE-PLD activity in high-throughput screening assays (i.e., PEDA1, PED6, and flame-NAPE) (21), all share a six-carbon *N*-acyl chain with an ω-dinitrophenyl moiety, a structure that shares some similarity to several previously reported *N*-carboxyacyl-PE species of NALPEs (Fig. 1). Third, that PE where the aldehyde 15-E_2_-IsoLG modifies the headgroup nitrogen (*N*-IsoLG-PE) is a substrate for NAPE-PLD (22).

**Fig. 1:**
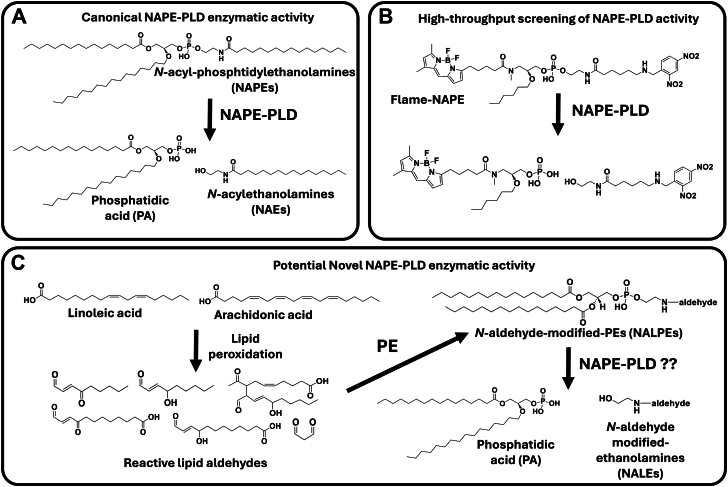
Canonical and hypothesized NAPE-PLD substrates. A: NAPE-PLD hydrolyzes its canonical substrates, NAPEs, to PA and *N*-acyl-ethanolamines. B: High-throughput screening for modulators of NAPE-PLD activity utilize fluorogenic probes such as flame-NAPE (pictured), PED6, and PED-A1, which have an *N*-acyl-nitrophenyl group as the quencher moiety to suppress BODIPY fluorescence. NAPE-PLD efficiently hydrolyzes these substrates to BODIPY-PA and *N*-acyl-nitrophenyl-ethanolamine suggesting that NAPE-PLD readily accommodates polar groups at the ω terminus of the *N*-acyl chain. C: Lipid peroxidation of PUFAs including linoleic acid and arachidonic acid leads to formation of reactive aldehydes, such as MDA, ONE, HNE, KODA, HODA, and IsoLG, and these reactive aldehydes can react with the headgroup nitrogen of PE to form NALPEs. The present study tests whether these NALPEs are also efficient substrates of NAPE-PLD.

To test our hypothesis that NAPE-PLD efficiently hydrolyzes NALPEs in addition to its canonical substrates (e.g., NAPEs), we further characterized the major species of NALPEs generated by lipid peroxidation and then tested various synthetic NALPEs for their ability to be hydrolyzed by recombinant NAPE-PLD. We found that many different species of NALPEs are hydrolyzed by NAPE-PLD, some with equivalent efficiency as canonical NAPEs. These studies suggest that NAPE-PLD may be an important degradative enzyme of NALPEs in vivo, so that its reduced expression during cardiometabolic diseases might result in markedly increased NALPE levels and thereby contribute to inflammation.

## Materials and Methods

### Materials and reagents

1,2-Dipalmitoyl-*sn*-glycero-3-phosphoethanolamine (dpPE), 1,2-dihexanoyl-*sn*-glycero-3-phosphoethanolamine (dhPE), *N*-glutaryl-PE, *N*-dodecanoyl-PE (renamed *N*-carboxyundecanoyl-PE [*N*-CUDA-PE]) were purchased from Avanti Polar Lipids (Alabaster, AL). *N*-Palmitoyl-(dipalmitoyl)PE (*N*-palmitoyl-PE) and *N*-linoleoyl-(dipalmitoyl)PE (*N*-linoleoyl-PE) were purchased from Echelon Biosciences (Salt Lake City, UT). Linoleic acid and arachidonic acid were purchased from Cayman Chemical (MI). 1,1,3,3-Tetramethoxypropane was purchased from Sigma-Aldrich, and 2,5-dimethyltetrahydrofuran was purchased from Thermo scientific. V70 (azobis[4-methoxy-2.4-dimethyl valeronitrile]) was purchased from Wako Chemicals. Organic solvents, including methanol, chloroform, dichloromethane, and acetonitrile, were HPLC grade from EMD Millipore.

### Oxidation of arachidonic acid/linoleic acid in the presence of PE

To investigate potential modifications of PE by reactive aldehydes resulting from lipid peroxidation, arachidonic acid and linoleic acid (10 mM each) were oxidized using 0.3 mM V70 as a free radical initiator in the presence of 0.5 mM PE in a reaction buffer of triethylamine acetate and isopropanol (1:1 ratio), incubating at 37°C overnight with ample oxygen. The lipid was extracted using the 2:1 chloroform/methanol (v/v), dried under nitrogen gas, and dissolved in mobile phase A for LC/MS injection. The control sample was prepared using the same process but without V70. Another set of samples was also prepared where dhPE was substituted for dpPE.

### Synthesis of *N*-aldehyde-modified PEs

IsoLG, KODA, ONE, and HNE were synthesized as previously described (23, 24). Butane dialdehyde (BDA) and MDA were freshly prepared by acidic conversion of 2,5-dimethyltetrahydrofuran and 1,1,3,3-Tetramethoxypropane, respectively, as previously described (25). Individual NALPEs were generated by reaction of aldehyde with 250 μM of dpPE overnight in 500 μl of reaction buffer (triethylamine acetate/isopropanol 1:1, v/v). The molar ratio of each aldehyde to PE was based on pilot studies to determine the amount of aldehyde needed to maximize yield of NALPEs. Final reactions used the following molar equivalents of aldehyde to PE: 3:1 BDA, 16:1 MDA; 10:1 HNE, 3:1 ONE, 5:1 KODA, and 2:1 IsoLG.

Reactions were extracted with 2:1 chloroform/methanol (v/v), and the yield of each product estimated based on LC/MS, with total yield based on reduction in PE and individual yield of individual species based on peak heights. Under these conditions, we estimated the amount of unmodified PE remaining after the reaction to be <50%, although all reactions had some unmodified PE remaining. For the reactions with HNE, ONE, and KODA, as well as the commercial preparation of *N*-CUDA-PE, we performed product ion scanning with the Q Exactive instrument using as precursor ions the major new products resulting from the reaction.

### LC/MS analysis of NALPEs

One set of LC/MS analysis to identify NALPEs formed during lipid peroxidation and analysis of synthetic NALPEs was performed using a Thermo Q-Exactive Orbitrap mass spectrometer coupled to a Dionex UPLC system (Thermo, San Jose, CA) using a 50 × 2.1 mm C18 Kinetix column (2.6 μm) directly coupled to the mass spectrometer with a constant flow rate of 0.1 ml/min. Analysis of synthetic NALPEs used the same chromatographic conditions and settings. Mobile phase A consisted of a mixture containing isopropanol, methanol, and water (in a ratio of 5:1:4, v/v/v), enriched with 0.2% formic acid and 0.66 mM ammonium formate, and mobile phase B was composed of isopropanol with 0.2% formic acid. For analysis of dpPE samples, a flow rate of 0.100 ml/min was applied, beginning with 5% mobile phase B held for 0.5 min, followed by a linear gradient to 95% B from 0.5 to 2.2 min. The gradient was held at 95% B until 4.1 min, then reduced back to 5% B by 5.0 min, and maintained at 5% B until 7.0 min. For analysis of dhPE samples, the gradient began with 5% B held until 0.5 min, increased to 95% B by 2.2 min, held at 95% B until 3.8 min, reduced back to 5% B by 4.5 min, and maintained at 5% B until 5.0 min. The column temperature was maintained at 40°C. ESI was performed using a heated ESI source with a spray voltage of 3,500 V, sheath gas set to 40, capillary temperature at 320°C, S-lens RF level set to 50, and the scan rate was 6 Hz. ThermoXcalibur Qual Browser software was used for data processing. For the reconstructed single-ion monitoring chromatographs, the plot type was set to mass range and the mass range entered as the calculated exact mass to the fourth decimal place, with 13-point Gaussian smoothing and 3 ppm mass tolerance. To identify the probable chemical composition of individual masses detected in the total ion current chromatograph and spectrum, the possible mass shift was calculated from the relevant charged unmodified PE mass ([M + H]+ or [M + H + NH3]+ for positive ion mode and [M - H]- or [M - H + NH3]- for negative ion mode), and this value entered into elemental composition calculator v1.0 at http://rna.rega.kuleuven.be/masspec/elcomp.htm with the following restrictions imposed: target mass ± 5 ppm, maximum carbon # = 22, maximum hydrogen # = 50, maximum nitrogen # = 1, maximum sulfur # = 0, maximum phosphate # = 0, nitrogen rule of even mass = even number of nitrogens, charge = 0. In most cases, the application returned only a single possible composition for an entered mass. If multiple possible compositions were returned, in every case, all but one composition could be eliminated because of the composition requiring more than two hydrogens or oxygens per carbon atom. In the case where the application returned no values for chemical composition, this was interpreted to indicate that the detected mass likely did not arise from an *N*-modified PE (e.g., was either a contaminant of the solvent system or an oxidized lipid product that did not modify PE). A summary of the detected modifications, the chemical composition, and the difference (in parts per million, ppm) between the observed and calculated mass is provided in supplemental Table S1.

LC/MS analyses were also performed using ThermoFinnigan Quantum ESI triple-quadrupole mass spectrometer equipped with a surveyor autosampler operating in positive mode. For analysis of NALPEs formed during lipid peroxidation, product mixtures were injected and separated on gradient HPLC using a 50 × 2.1 mm C18 Kinetix column (2.6 μm) directly coupled to the mass spectrometer with a constant flow rate of 0.1 ml/min. Mobile phase A consisted of a mixture containing isopropanol, methanol, and water (in a ratio of 5:1:4, v/v/v), enriched with 0.2% formic acid, 0.66 mM ammonium formate, and 0.003% phosphoric acid. Mobile phase B composed of isopropanol with 0.2% formic acid. Starting conditios were 5% mobile phase B and held for 0.5 min, followed by a gradient ramp to 95% mobile phase B over 2 min, which was then held for another 2 min before a rapid transition back to 5% mobile phase B over 0.5 min and the column allowed to re-equilibrate for 2 min prior to the next injection. ESI was performed at 3,300 V, and the ion-transfer tube operated at 25 V and 270°C. The tube lens voltage was set to −180 V, and the scan rate was 17 Hz. Precursor scanning for reactions with dpPE was performed from *m/z* 350–1,500 Da using product ion *m/z* 551.5, and for reactions with dhPE was performed from *m/z* 150–1,200 Da using product ion *m/z* 271.2. A summary of the detected modifications is provided in supplemental Table S2.

For product ion scanning of synthesized NALPEs, direct line infusion of the reaction mixture into the Thermo Q-Exactive Orbitrap mass spectrometer was performed, with the instrument set to all ion fragmentation mode with a resolution of 15,000. Each NALPE precursor ion was isolated at its exact *m/z* with a 0.4 *m/z* isolation window. Fragmentation was done using five different high-energy collisional dissociation settings: 10, 15, 20, 30, 40, and 50. The automatic gain control target was set to 1 × 10^6^, and MS spectra were collected over a range from *m/z* 50 to just below the *m/z* of the precursor ion for each NALPE. A summary of the product ions generated is given in supplemental Table S3.

To quantify NALPE hydrolysis by NAPE-PLD hydrolysis, a 3.5-min HPLC run was performed using a 2.1 mm C18 guard column (Phenomenex AJ0-8782) with a flow rate of 100 μl/min. The product mixtures were injected with an injection volume of 2 μl and separated using the following gradient conditions: starting at 5% B for 0.5 min, increasing to 95% B and holding for 2.5 min, then returning to 5% B at 3.5 min. Multiple reaction monitoring was used to quantify the reaction (Table 1).

**Table 1:** Ions used for multiple reaction monitoring to quantify NAPE-PLD hydrolysis

NALPEs/NAPEs/PA	m/z [M + H]^+^ → *m/z* 551.5	m/z [M + NH_4_]^+^ → *m/z* 551.5
*N*-BDA-PE	742.5 (Pyrrole)	759.5 (Pyrrole)
*N*-MDA-PE	746.5 (SB)	763.5 (SB)
*N*-HNE-PE	812.6 (Pyrrole)	829.6 (Pyrrole)
	848.6 (Michael adduct)	865.6 (Michael adduct)
*N*-ONE-PE	828.6 (SB)	845.6 (SB)
	846.6 (KA)	863.6 (KA)
*N*-glutaryl-PE	806.5	823.5
*N*-CUDA-PE	904.6	921.6
*N*-KODA-PE	900.6 (SB)	917.6 (SB)
	918.6 (KA)	935.6 (KA)
*N*-IsoLG-PE	1006.7 (AL)	1023.7 (AL)
	1024.7 (Ltm)	1041.7 (Ltm)
*N*-palmitoyl-PE	930.7	947.7
*N*-linoleoyl-PE	954.7	971.7
Dipalmitoyl-PA	649.5	666.5

N-AL-ethanolamine (Etn)	*m/z* [M + H]^+^ → *m/z* 62	*m/z* [M + NH_4_]^+^ → *m/z* 62
*N*-BDA-Etn	112.1 (Pyrrole)	129.1 (Pyrrole)
*N*-MDA-Etn	116.1 (SB)	133.1 (SB)
*N*-HNE-Etn	182.2 (Pyrrole)	199.2 (Pyrrole)
	218.2 (Michael adduct)	235.2 (Michael adduct)
*N*-ONE-Etn	198.1 (SB)	215.2 (SB)
	216.2 (KA)	233.2 (KA)
*N*-glutaryl-Etn	176.1	193.1
*N*-CUDA-Etn	274.2	291.2
*N*-KODA-Etn	270.2 (SB)	287.2 (SB)
	288.2 (KA)	305.2 (KA)
*N*-IsoLG-Etn	376.2 (AL)	393.3 (AL)
	394.3 (Ltm)	411.3 (Ltm)
*N*-palmitoyl-Etn	300.3	317.3
*N*-linoleoyl-Etn	324.3	341.3

Internal standards	*m/z* [M + H]^+^ → *m/z* 603.5	m/z [M + NH_4_]^+^ → *m/z* 603.5
Dioleoyl-PA	701.5	718.5
d_4_-NPPE	986.6	1003.6

### Expression and purification of NAPE-PLD

Recombinant mouse NAPE-PLD was expressed in *Escherichia coli* as previously described with slight modifications (22). The purification process involved lysis of induced bacterial cultures in lysis buffer (50 mM NaH_2_PO_4_, 300 mM NaCl, 10 mM imidazole, pH 8.0), incubation with TALON Superflow agarose beads contain cobalt ion, elution using elution buffer (50 mM NaH_2_PO_4_, 300 mM NaCl, 250 mM imidazole, pH 8.0), and washing with wash buffer (50 mM NaH2PO4, 300 mM NaCl, 20 mM imidazole, pH 8.0). Purified fractions were concentrated, dialyzed against buffer (Hanks' balanced salt solution), and stored at −80°C.

### Hydrolysis of NALPEs by NAPE-PLD

To test the ability of NAPE-PLD to hydrolyze individual NALPEs, a reaction solution was prepared with the following reagents: 15 μM of phospholipid (PE + aldehyde reaction mixture), 0.4% *N*-octyl glucoside, 50 mM Tris-HCl, and 20 ng/ml NAPE-PLD. The reaction was performed for each NALPE separately by incubation at 37°C for 2 h and was quenched by extraction using a 2:1 chloroform/methanol mixture containing dioleoyl-phosphatidic acid (PA) and d_4_-*N*-palmitoyl-(dioleoyl)PE as internal standards. The organic layer was isolated and evaporated under nitrogen, followed by reconstitution in mobile phase A for analysis by LC/MS analysis using the instrument in multiple reaction monitoring mode (Table 1). To compare the relative rate of hydrolysis of NAPE-PLD substrates directly, approximately equal concentrations (based on signal intensity in triple quadruple mass spectrometer) of *N*-MDA-PE, *N*-BDA-PE, *N*-HNE-PE, *N*-ONE-PE, *N*-CUDA-PE, *N*-KODA-PE, *N*-IsoLG-PE, *N*-palmitoyl-PE, and *N*-linoleoyl-PE were combined together to create a substrate mixture. This substrate mixture was incubated with active enzyme and aliquots withdrawn after 1, 2, and 3 h of incubation. As a control, separate aliquots of the mixture were also incubated with heat-inactivated enzyme and processed in a similar manner. Samples incubated with inactive enzyme were used to establish the starting levels of each lipid without hydrolysis (0 h with active enzyme).

## Results

### Identification of major NALPE species formed during lipid peroxidation in vitro

**Fig. 2:**
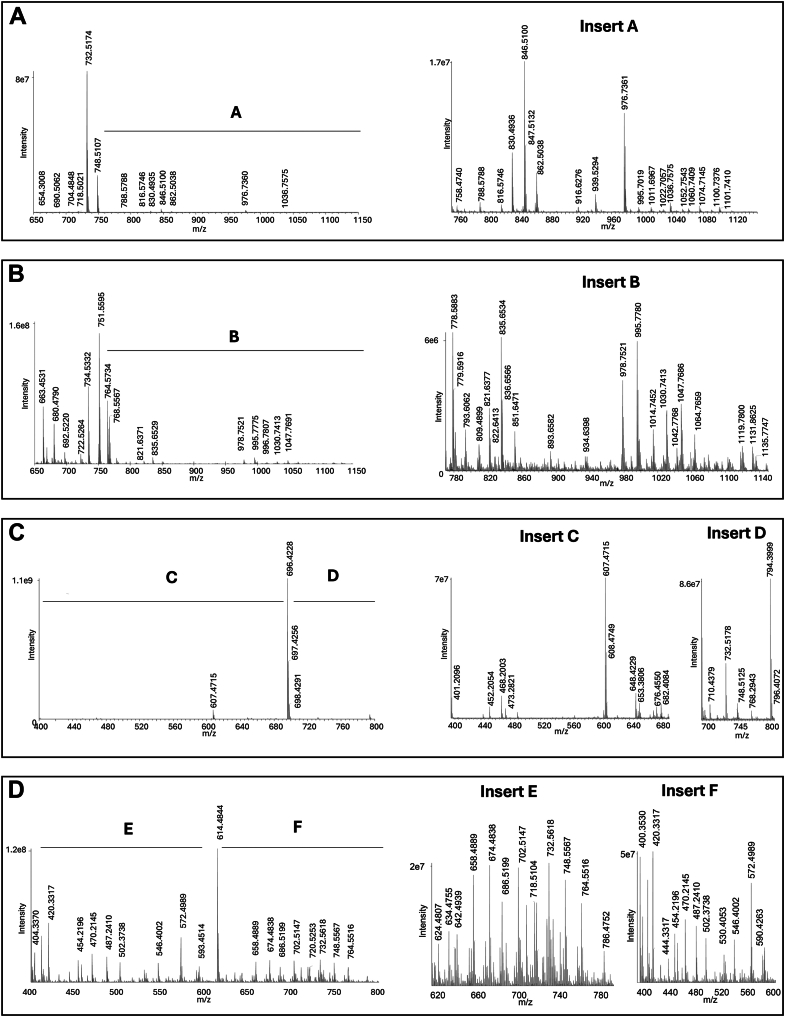
Mass spectrometric detection of multiple NALPEs generated by lipid peroxidation. Arachidonic acid and linoleic acid were oxidized in the presence of dpPE or dhPE by addition of V70 as a radical initiator. The resulting products were analyzed using an Exactive Orbitrap mass spectrometer in both positive and negative ion scanning modes. The mass spectrum of the broad peak(s) observed in the total ion current chromatogram for the oxidized samples is shown, with inserts providing magnified views of specific regions of the spectrum: A: dpPE negative ion mode; B: dpPE positive ion mode; C: dhPE negative ion mode; and D: dhPE positive ion mode.

**Fig. 3:**
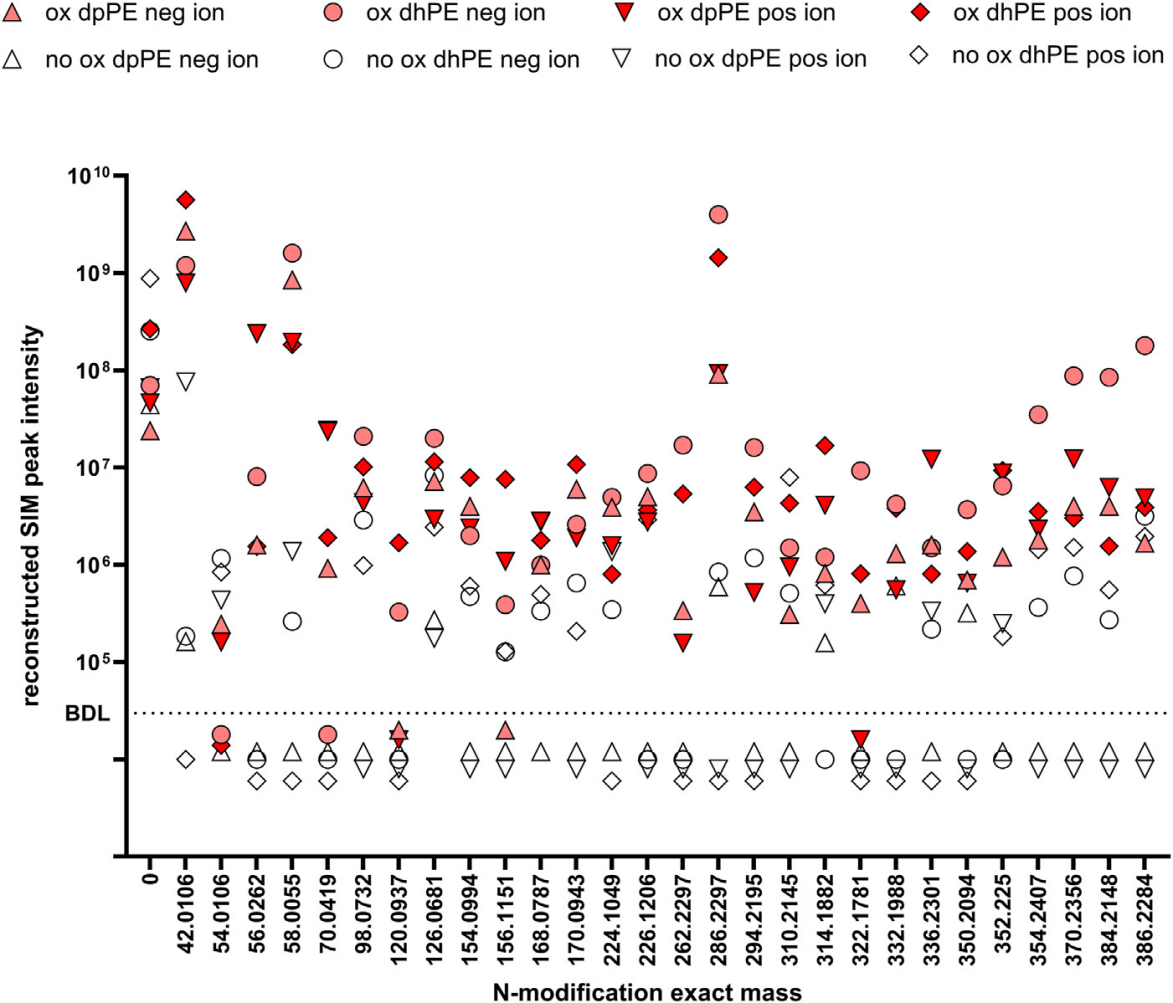
Relative abundance of the major NALPE species generated by lipid peroxidation. The relative intensity of peaks found for the reconstructed single-ion monitoring (SIM) chromatographs for each of the potential NALPEs (classified by the increase in mass from unmodified PE) from the same mass spectral analysis shown in Figure 2 from both the positive ion (pos ion) and negative ion (neg ion) analysis of reactions with (ox) or without (no ox) V70 added to the mixture of linoleic acid and arachidonic acid with either dpPE or dhPE. Modification with reconstructed SIM peak intensity <1 × 10^5^ cps is indicated as being below the detection limit (BDL).

**Fig. 4:**
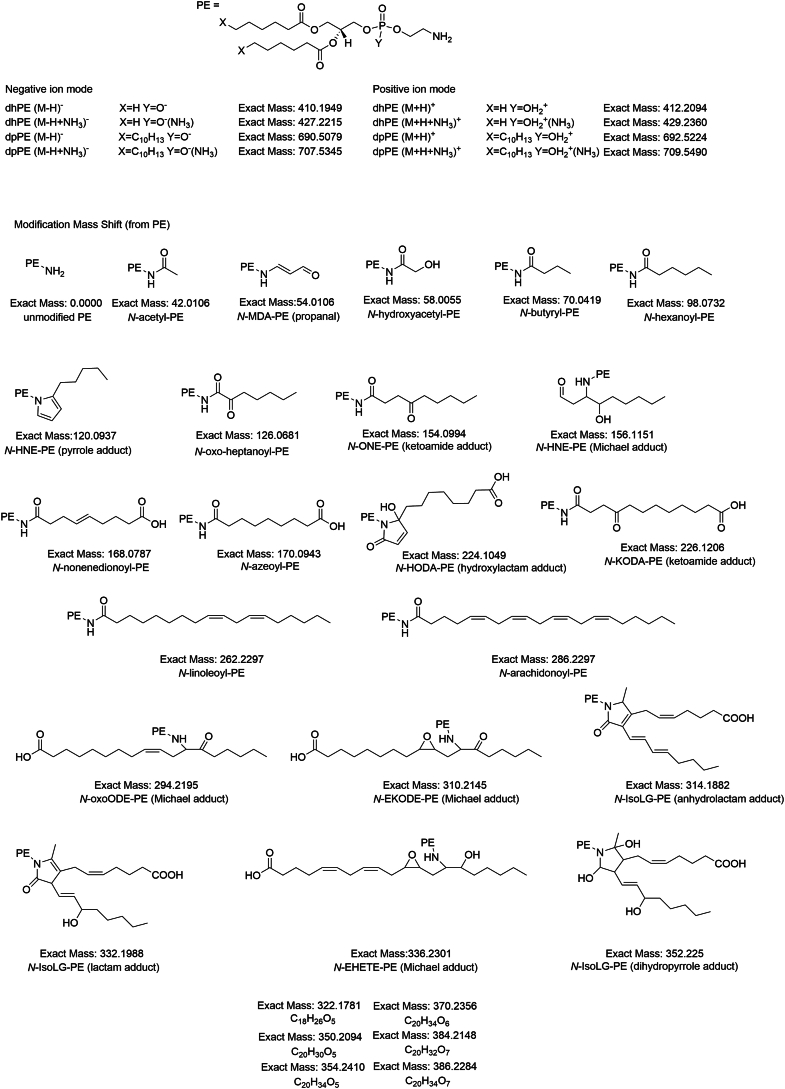
Proposed structures of *N*-modification for NALPE species generated by lipid peroxidation. The exact increase in mass (from unmodified PE) as well as the proposed structure of each *N*-modification for individual NALPEs is shown, along with the exact mass and structure of dpPE and dhPE. For NALPE species where a likely structure could not be easily rationalized, only the predicted chemical composition is shown.

**Fig. 5:**
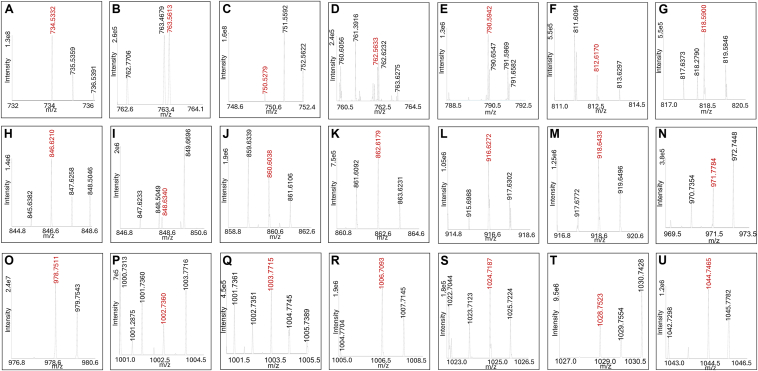
Representative limited mass spectrum for each NALPE. Limited mass spectrum is from the positive ion analysis of arachidonic acid and linoleic acid oxidized in the presence of dpPE. Calculated *m/z* values listed after each NALPE below are for the [M + H]+ ion, except for *N*-MDA-PE (propanal adduct), which is the [M + H + NH_3_]+ ion. A: *N*-acetyl-PE, *m/z* 734.5330; B: *N*-MDA-PE (propanal adduct), *m/z* 763.5596; C: *N*-hydroxy-acetyl-PE, *m/z* 750.5279; D: *N*-butyryl-PE, *m/z* 762.5643; E: *N*-hexanoyl-PE, *m/z* 790.5956; F: *N*-HNE-PE (pyrrole adduct), *m/z* 812.6161; G: *N*-oxo-heptanoyl-PE, *m/z* 818.5905; H: *N*-ONE-PE (KA adduct), *m/z* 846.6218; I: N-HNE-PE (Michael adduct), *m/z* 848.6375; J: *N*-nonenedionoyl-PE, *m/z* 860.6011; K: *N*-azeoyl-PE (*N*-nonanedionoyl-PE) *m/z* 862.6167; L: *N*-HODA-PE, *m/z* 916.6273; M: *N*-KODA-PE (KA adduct), *m/z* 918.6430; N: *N*-linoleoyl-PE, *m/z* 954.7521; O: *N*-arachidonoyl-PE, *m/z* 978.7521; P: *N*-oxoODE-PE (Michael adduct), *m/z* 986.7419; Q: *N*-EKODE-PE (Michael adduct), *m/z* 1002.7369; R: *N*-IsoLG-PE (AL adduct) *m/z* 1006.7106; S: *N*-IsoLG-PE (Ltm adduct), *m/z* 1024.7212; T: *N*-EHETE-PE, *m/z* 1028.7525; and U: *N*-IsoLG-PE (dihydropyrrole adduct), *m/z* 1044.7474.

To identify the major NALPEs that could form during lipid peroxidation, we oxidized arachidonic acid and linoleic acid in the presence of dpPE, an endogenous PE. We also performed the same reaction with a model PE, dhPE, which is more water soluble. Products generated in both these reactions were compared with the control reactions without oxidant by two LC/MS approaches: mass scanning using a high-resolution Orbitrap mass spectrometer and parent mass scanning using a triple quadrupole mass spectrometer. We utilized both positive and negative ion modes for the high-resolution mass scanning approach (four total analyses of oxidation samples and four total analyses of unoxidized samples). Comparison of the total ion chromatographs between the oxidized versus nonoxidized samples in each mode showed chromatographic peaks unique to the oxidized samples (supplemental Fig. S1). The mass spectrum from each of the four analyses of oxidized samples is shown in Fig. 2. To identify equivalent N-modifications in each analysis, we calculated the mass difference (mass shift) between each detected ion peak versus the ion peaks of the original PEs and used this to determine the likely chemical formula of the compound. A total of 28 mass shifts representing potential NALPE*s* were consistently detected (based on having a reconstructed single-ion monitoring peak intensity ≥10^5^ counts per second) in at least two of the four oxidized samples (Fig. 3) and a tentative structure assigned based on previously reported modifications or reactions with known aldehyde precursors that could plausibly give rise to the mass shift (Fig. 4). Representative limited mass spectrum from the positive ion analysis of the dpPE oxidation sample for each modification with a putative structure is shown in Fig. 5. The observed exact mass for each modification in all four analyses is listed in supplemental Table S1.

As an additional confirmation of NALPE formation, similar oxidation mixtures were analyzed using a triple quadrupole mass spectrometer operating in positive ion parent scanning mode, with the product ion set to match the diacylglycerol portion of the relevant PE (*m/z* 551.5 for dpPE reaction and *m/z* 271.2 for dhPE reaction). The spectrum of peaks generated by these analyses is shown in supplemental Figs. S2 and S3.

Tentative structures for most of the modifications found in the oxidation reactions could be rationalized based on previously identified aldehyde species and their adducts. The +42.0106 amu, +58.055 amu, +70.0419 amu, and +98.0732 amu modification are consistent with previously reported *N*-acetyl-PE, *N*-hydroxyacetyl-PE, *N*-butyryl-PE, and *N*-hexanoyl-PE, respectively (Fig. 5) (11, 26, 27). 4-hydroxyalkenals such as HNE and HODA are known to form pyrrole and oxidized pyrrole adducts, as well as Michael adducts (9, 28, 29) and the +120.0937 amu and +224.1049 amu modifications are consistent with the pyrrole adduct species of *N*-HNE-PE and the oxidized pyrrole (hydroxylactam) adduct species of *N*-HODA-PE, respectively, whereas the +156.1151 amu modification is consistent with the *N*-HNE-PE Michael adduct. 4-ketoalkenal such as ONE generates 4-ketoamide (KA) adducts when reacted with lysine (28), and the +154.0994 amu and +226.1206 amu modifications are consistent with the 4-KA adduct species of *N*-ONE-PE and *N*-KODA-PE, respectively. ONA is a well-characterized Hock cleavage product formed during linoleic acid peroxidation (7), whereas 9-oxo-non-5-enoic acid is a predicted minor product of arachidonic acid peroxidation. The +168.0787 amu and +170.0943 amu modifications are consistent with *N*-nonenedionyl-PE and *N*-azeoyl-PE, which would be expected to arise from oxidation of the SB adducts of 9-oxo-non-5-enoic acid and ONA, respectively. 4-ketoaldehydes such as IsoLG give rise to dihydropyrrole adducts that undergo dehydration to pyrrole adducts, which then undergo further oxidation to lactam (Ltm) and hydroxylactam adducts (23, 30), and the +314.1882, +332.1988, and +352.2250 amu modifications are consistent with the anhydrolactam (AL), Ltm, and dihydropyrrole species of *N*-IsoLG-PE, respectively. Peroxidation of linoleic acid has previously been described to generate 9- and 13-oxo-octadecadienoic acid as well as 9-epoxy-13-keto-octadecaenoic acid and 9-keto-13-epoxy-octadecaenoic acid. Peroxidation of arachidonic acid can generate various epoxy-hydrodroxy-eicosatrienoic acids. The +294.2195, +310.2145, and +336.2301 amu modifications can be rationalized as Michael adduct species of *N*-oxo-octadecadienoic acid-PE, *N*-epoxy-octadecaenoic acid-PE, and *N*-epoxy-hydrodroxy-eicosatrienoic acid-PE, respectively.

For some modifications, a plausible mechanism that might give rise to the modification could not be easily rationalized. For example, the oxidized samples showed a relatively high abundance of +262.2297 amu and +286.2297 amu modifications compared with unoxidized samples (Fig. 3), consistent with formation of *N*-linoleoyl-PE and *N*-arachidonoyl-PE (Fig. 4), respectively. However, the formation of these NAPEs is surprising given that the formation of amides from the reaction of fatty acids with primary amines is typically of low yield in the absence of catalyst, although it is possible that peroxidation of fatty acids to peroxyacids might facilitate the formation of NAPEs. There were also modifications for which either a reasonable structure of the reactive lipid precursor and/or the final product is not readily apparent. For instance, the +322.1781 amu modification is consistent with an 18 carbon molecules with five oxygens and the +350.2094, +354.2410, +370.2356, +384.2148, and +386.2284 amu modifications are consistent with 20 carbon molecules that include five to seven oxygens (Fig. 4). Finally, we anticipated that a +54.0106 amu modification consistent with the propenal adduct of *N*-MDA-PE would be markedly elevated in the oxidized samples, but this was not the case (Fig. 3). It is possible that this is because *N*-MDA-PE is produced by autooxidation even in the absence of oxidizer.

### Synthesis of NALPEs for hydrolysis studies

To confirm the identity of some of the putative NALPE species (e.g., *N*-KODA-PE) and to test whether individual NALPEs are robust substrates for NAPE-PLD, we reacted dpPE with synthesized versions of major endogenous aldehydes or their analogs, characterized the major products of these reactions using high-resolution mass scanning in positive ion mode and determined whether these synthetic NALPEs were substrates for hydrolysis by NAPE-PLD. We did not synthesize or test *N*-acetyl-PE, *N*-butyryl-PE, and *N*-hexanoyl-PE, as these NAPEs have previously been tested as substrates for NAPE-PLD, with both *N*-butyryl-PE and *N*-hexanoyl-PE being readily hydrolyzed (16).

**Fig. 6:**
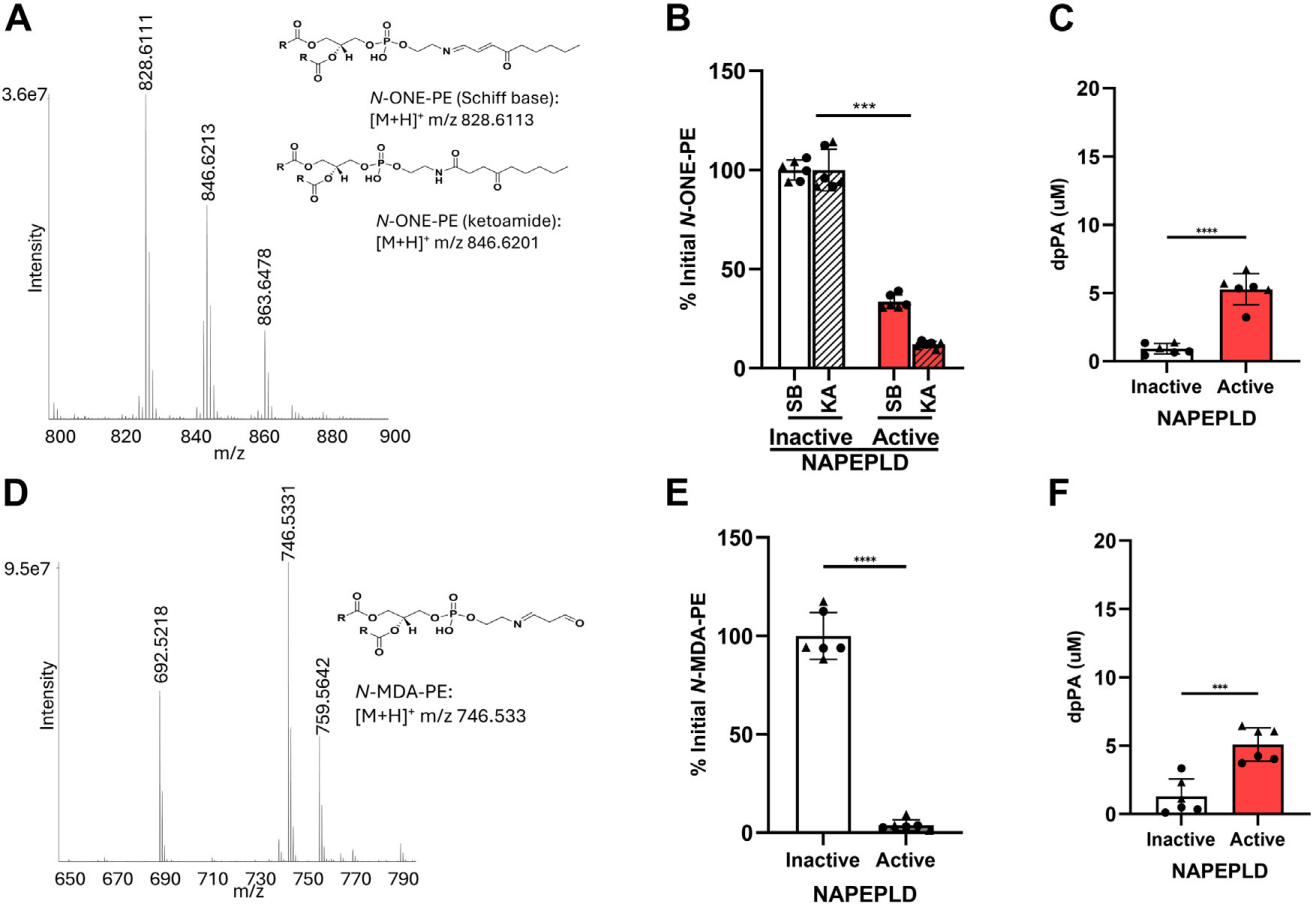
*N*-ONE-PE and *N*-MDA-PE are hydrolyzed by NAPE-PLD. A: Dipalmitoyl-PE ([M + H]+ *m/z* 692.5224) was reacted with ONE, and the resulting products were analyzed using Q Exactive Orbitrap mass spectrometer in positive ion scanning mode. The putative structure of the two major *N*-ONE-PE products, SB adduct and KA adduct, is shown. Active recombinant NAPE-PLD or heat-inactivated enzyme was incubated with the *N*-ONE-PE mixture for 2 h and the changes in *N*-ONE-PE species (B) and dipalmitoyl-phosphatidic acid (dpPA) (C) levels determined by LC/MS. The initial level of each *N*-ONE-PE species was set as the amount detected in the sample with inactive enzyme. D: Dipalmitoyl-PE was reacted with MDA, and the resulting *N*-MDA-PE products were analyzed in a similar manner as *N*-ONE-PE. The putative structure of the major *N*-MDA-PE product is shown. *N*-MDA-PE was incubated with active or inactive NAPE-PLD for 2 h, and the resulting change in levels of *N-*MDA-PE (E) and dpPA (F) monitored by LC/MS in a similar manner as for *N*-ONE-PE. For each, mean ± SEM is shown with Student’s *t*-test comparison of active versus inactive enzyme, ∗∗∗*P* < 0.001 and ∗∗∗∗*P* < 0.0001.

We considered the putative KA adduct of *N*-ONE-PE as the most likely NALPE to be a substrate of NAPE-PLD, as it only differs from the canonical substrates of NAPE-PLD, NAPEs, by the addition of a ketone group at carbon 4. Reaction of ONE with dpPE yielded two major species, one consistent with the predicted KA adduct (*m/z* 846.6213) and the other consistent with the predicted SB adduct (*m/z* 828.6111) (Fig. 6A and supplemental Fig. S4A). Product ion scanning of these two precursor ions primarily generated product ions from the dipalmitoyl PE moiety including fragmentation before the proximal phosphodiester bond (*m/z* 551.5035); from the dipalmitoyl O-acyl chains (*m/z* 57.0700, 67.9542, 71.0854, 81.0699, 85.1012, 95.0856, 102.1276, 109.1013, 123.1169, 137.1318, and 239.2363); and from fragmentation after the distal phosphodiester bond (*m/z* 198.1489 and *m/z* 180.1382 for the KA and SB, respectively); but several minor product ions derived from the headgroup are consistent with the proposed adduct structure (supplemental Figs. S5 and S6). Incubation of this reaction mixture containing both *N*-ONE-PE species with active NAPE-PLD for 2 h resulted in >90% reduction in signal for the *N*-ONE-PE KA species and about a 65% reduction in signal for the *N*-ONE-PE SB species, compared with the signal obtained when the *N*-ONE-PE mixture was incubated with heat-inactivated enzyme (Fig. 6B and supplemental Fig. S4B). Incubation with active enzyme also produced 5.34 μM PA, consistent with a phospholipase D-type reaction being the major reaction by which NAPE-PLD degraded *N*-ONE-PE (Fig. 6C and supplemental Fig. S4C). We were also able to detect the expected *N*-ONE-ethanolamine KA product (supplemental Fig. S7A) in the reaction with active enzyme, although the signal was quite weak, most likely because the extraction conditions and LC/MS instrument setting for this relatively hydrophilic product (compared with parent NALPE and PA) were not optimal.

Given the prominent formation of an SB adduct with *N*-ONE-PE, we chose to further explore the effect of this alternative linkage compared with the amide bond on the ability of NAPE-PLD to hydrolyze substrate. Reaction of MDA with PE generated a product of *m/z* 746.5331, consistent with the SB (propanal) species of *N*-MDA-PE (Fig. 6D and supplemental Fig. S4D). Incubation of *N*-MDA-PE with active NAPE-PLD for 2 h resulted in essentially complete loss of the *N*-MDA-PE signal (Fig. 6E and supplemental Fig. S4E) and produced 6.45 μM PA (Fig. 6F and supplemental Fig. S4F). We were unable to detect the *N*-MDA-ethanolamine product, which was not unexpected given its relative hydrophilicity.

**Fig. 7:**
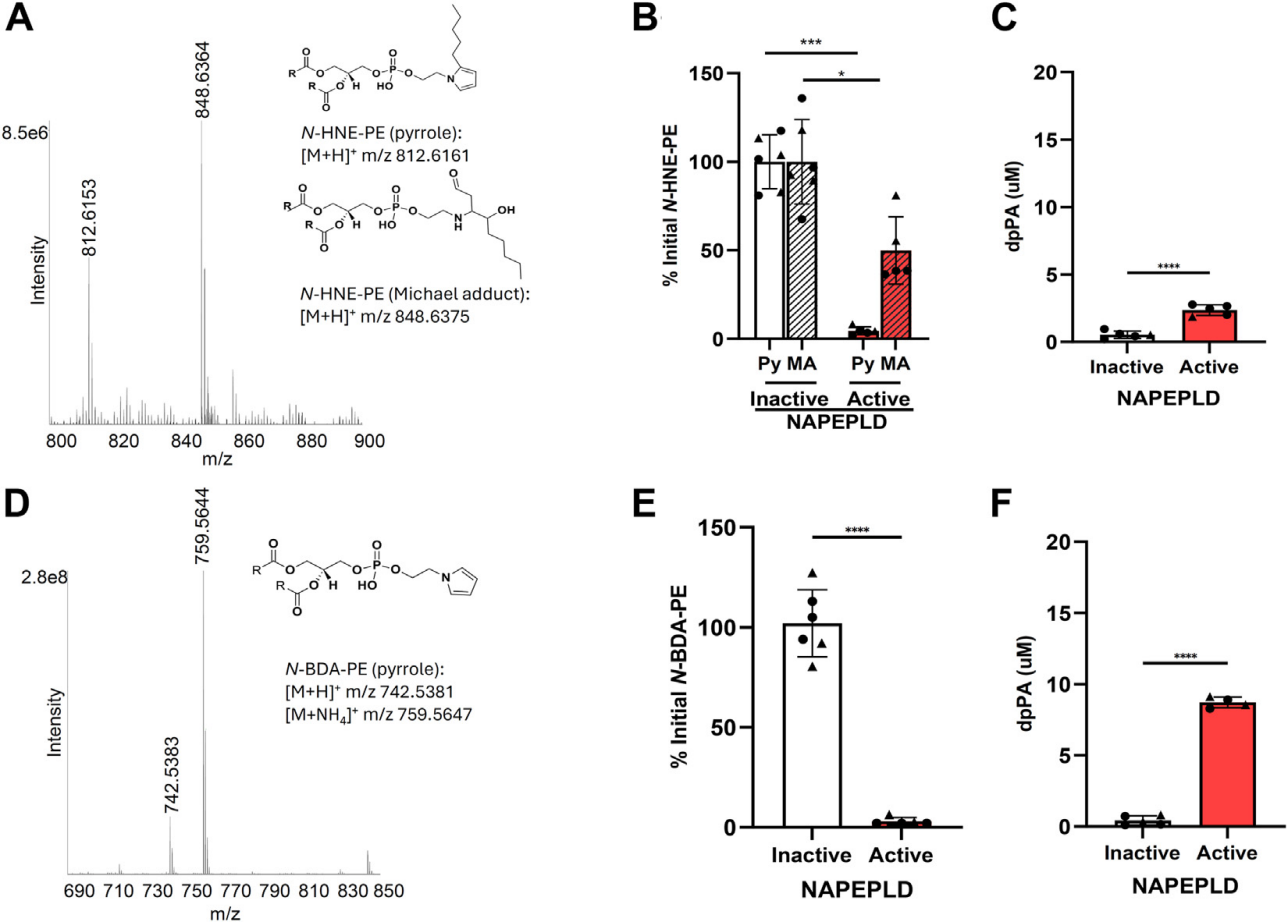
*N*-HNE-PE and *N*-BDA-PE are hydrolyzed by NAPE-PLD. A: Dipalmitoyl-PE was reacted with HNE, and the resulting products were analyzed using Q Exactive Orbitrap mass spectrometer in positive ion scanning mode. The putative structures of the two major *N*-HNE-PE products, pyrrole (Py) and Michael adduct (MA), are shown. Active or heat-inactivated NAPE-PLD were incubated with *N*-HNE-PE for 2 h and changes in levels of *N*-HNE-PE (B) and dipalmitoyl-PA, PA (C) determined by LC/MS. D: Dipalmitoyl-PE was reacted with BDA, and the resulting reaction products were analyzed by LC/MS. The putative structure of the major *N*-BDA-PE product is shown. E and F: *N*-BDA-PE was incubated with active or inactive NAPE-PLD, and the resulting changes in the levels of *N*-BDA-PE (E) and dpPA (F) were measured by LC/MS. For each, mean ± SEM is shown with Student’s *t*-test comparison of active versus inactive enzyme, ∗*P* < 0.05∗∗∗*P* < 0.001, and ∗∗∗∗*P* < 0.0001. dpPA, dipalmitoyl-phosphatidic acid.

HNE only differs from ONE by the addition of two hydrogens, but HNE is known to form distinct adducts from that of ONE. Reaction of HNE with PE yielded species consistent with the pyrrole (*m/z* 812.6153) and Michael addition adducts (*m/z* 848.6363) (Fig. 7A and supplemental Fig. S8A). Major product ions seen during product ion scanning of these two precursor ions include those from fragmentation before the proximal (*m/z* 551.5035) and after the distal phosphodiester bond (*m/z* 164.1439 and m/z 200.1645, for the pyrrole and Michael adduct species, respectively) (supplemental Figs. S9 and S10). Incubation of *N*-HNE-PE with active NAPE-PLD for 2 h resulted in essentially complete loss of the *N*-HNE-PE pyrrole signal, about 50% loss of the *N*-HNE-PE Michael adduct signal (Fig. 7B and supplemental Fig. S8B), and produced 2.48 μM PA (Fig. 7C and supplemental Fig. S8C).

To further investigate the ability of NAPE-PLD to hydrolyze substrates with *N*-pyrrole linkages, we reacted BDA (also known as succinaldehyde) with PE and characterized the resulting products. This reaction generated products of *m/z* 742.5383 and *m/z* 759.5644, consistent with the [M + H]^+^ and [M + NH_4_]^+^ ions for the *N*-pyrrole species (Fig. 7D and supplemental Fig. S8D). We noted that similar *N*-pyrrole adducts of lysine and DNA have been previously reported (31), but we did not detect signals consistent with the *N*-BDA-PE pyrrole in our oxidation mixture. Incubation of *N*-BDA-PE with active NAPE-PLD resulted in essentially complete hydrolysis of this pyrrole species (Fig. 7E and supplemental Fig. S8E) and produced 8.29 μM PA (Fig. 7F and supplemental Fig. S8F).

**Fig. 8:**
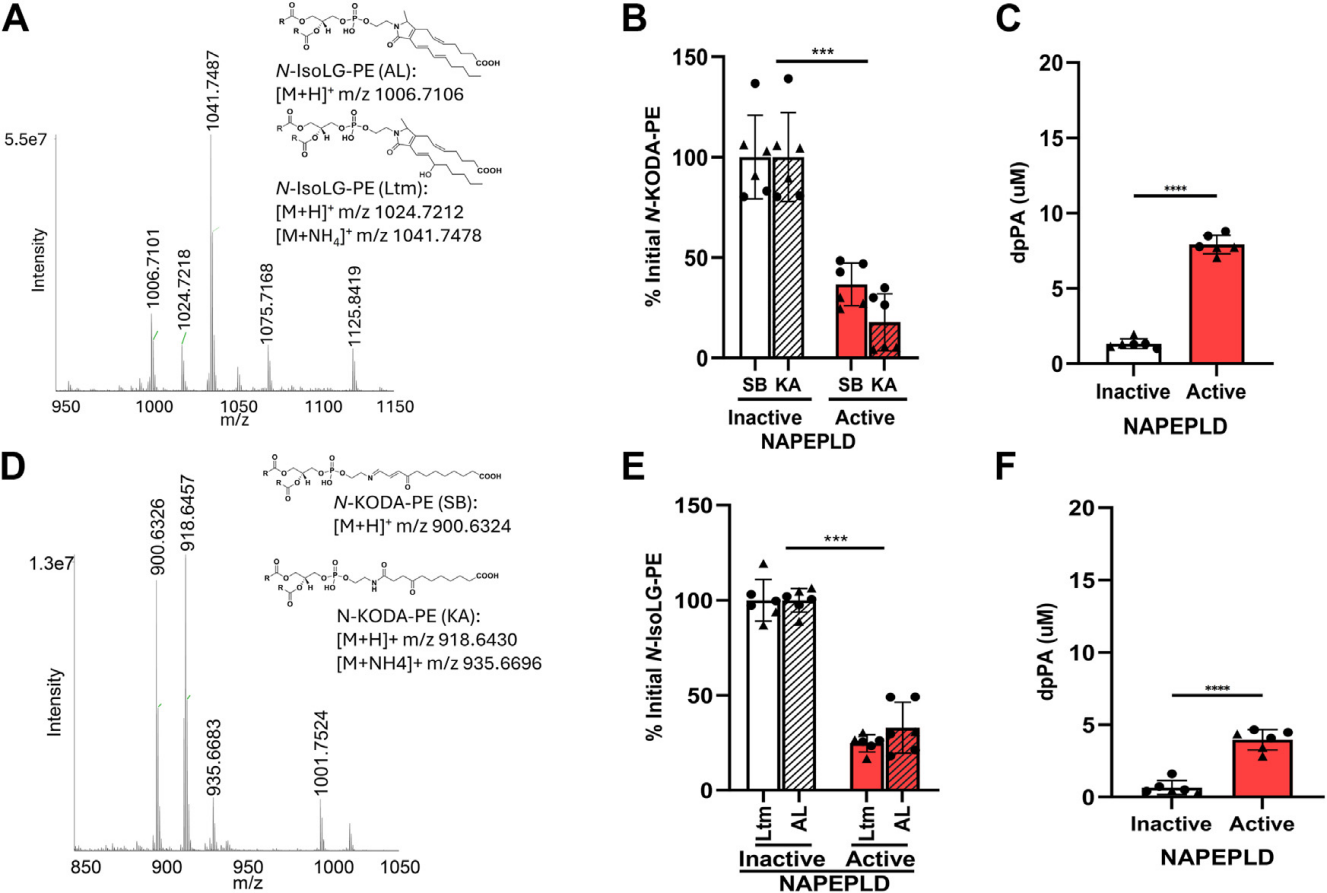
*N-*IsoLG-PE and *N*-KODA-PE are hydrolyzed by NAPE-PLD. A: Dipalmitoyl-PE was reacted with IsoLG, and the resulting products were analyzed using Q Exactive Orbitrap mass spectrometer in positive ion scanning mode. The putative structures of the major *N*-IsoLG-PE products, AL and Ltm, are shown. Active recombinant NAPE-PLD or heat-inactivated enzyme was incubated with *N*-IsoLG-PE for 2 h, and the resulting changes in the levels of *N*-IsoLG-PE (B) and dipalmitoyl-PA (dpPA) (C) were measured by LC/MS. D: Dipalmitoyl-PE was reacted with KODA, and the resulting products were analyzed by LC/MS in a similar manner as for *N*-IsoLG-PE. Active or heat-inactivated NAPE-PLD was incubated with *N*-KODA-PE for 2 h, and changes in levels of *N*-KODA-PE (E) and dpPA (F) were determined by LC/MS. For each, mean ± SEM is shown with Student’s *t*-test comparison of active versus inactive enzyme, ∗*P* < 0.05∗∗∗*P* < 0.001, and ∗∗∗∗*P* < 0.0001.

In each of the above reactions, the headgroup has either an alkyl tail or is entirely hydrophobic. However, a number of NALPEs retain the terminal carboxylate moiety of the original fatty acid from which the lipid aldehyde is produced. We had previously found that NAPE-PLD could hydrolyze *N*-IsoLG-PE, which has such a terminal carboxylate. To confirm our previous results, we reacted 15-E_2_-IsoLG with PE and characterized the resulting products. This reaction generated multiple adduct species, with the most prominent being those with *m/z* 1006.7101, *m/z* 1024.7218, and *m/z* 1041.7487, consistent with the formation of the AL and Ltm species of *N*-IsoLG-PE (Fig. 8A and supplemental Fig. S11A). When we incubated this *N*-IsoLG-PE mixture with NAPE-PLD for 2 h, we found that about 75% of both the AL and Ltm species were hydrolyzed (Fig. 8B and supplemental Fig. S11B) and produced 4.65 μM PA (Fig. 8C and supplemental Fig. S11C). We were also able to detect *N*-IsoLG-ethanolamine AL in the reaction with active enzyme, although the signal was relatively weak (supplemental Fig. S7B).

To further explore the effect of the terminal carboxylate moiety, we investigated whether *N*-KODA-PE is a substrate for NAPE-PLD. KODA is an analog of ONE that similarly results from the peroxidation of linoleic acid, except that it derives from fragmentation of the α-end of the molecule so that it retains the terminal carboxylate moiety. Reaction of KODA with PE yielded products whose masses were consistent with a KA adduct (*m/z* 918.6457) and an SB adduct (*m/z* 900.6326) (Fig. 8D and supplemental Fig. S11D). Major product ions generated during product ion scanning of these two precursor ions include those not only from fragmentation before the proximal (*m/z* 551.5035) and after the distal phosphodiester bond (*m/z* 270.1699 and *m/z* 252.1593 for KA and SB, respectively) but also from additional fragmentation at the ω-terminal carboxylate of the headgroup (*m/z* 224.1644 and *m/z 206.1540* for KA and SB, respectively) (supplemental Figs. S12 and S13). Incubation of *N*-KODA-PE with NAPE-PLD resulted in about 60% hydrolysis of the SB adduct and 80% hydrolysis of the KA adduct (Fig. 8E and supplemental Fig. S11E), with robust production of PA (8.47 μM) (Fig. 8F and supplemental Fig. S11F). We were also able to detect *N*-KODA-ethanolamine KA and SB in the reaction with active enzyme (supplemental Fig. S7C, D).

**Fig. 9:**
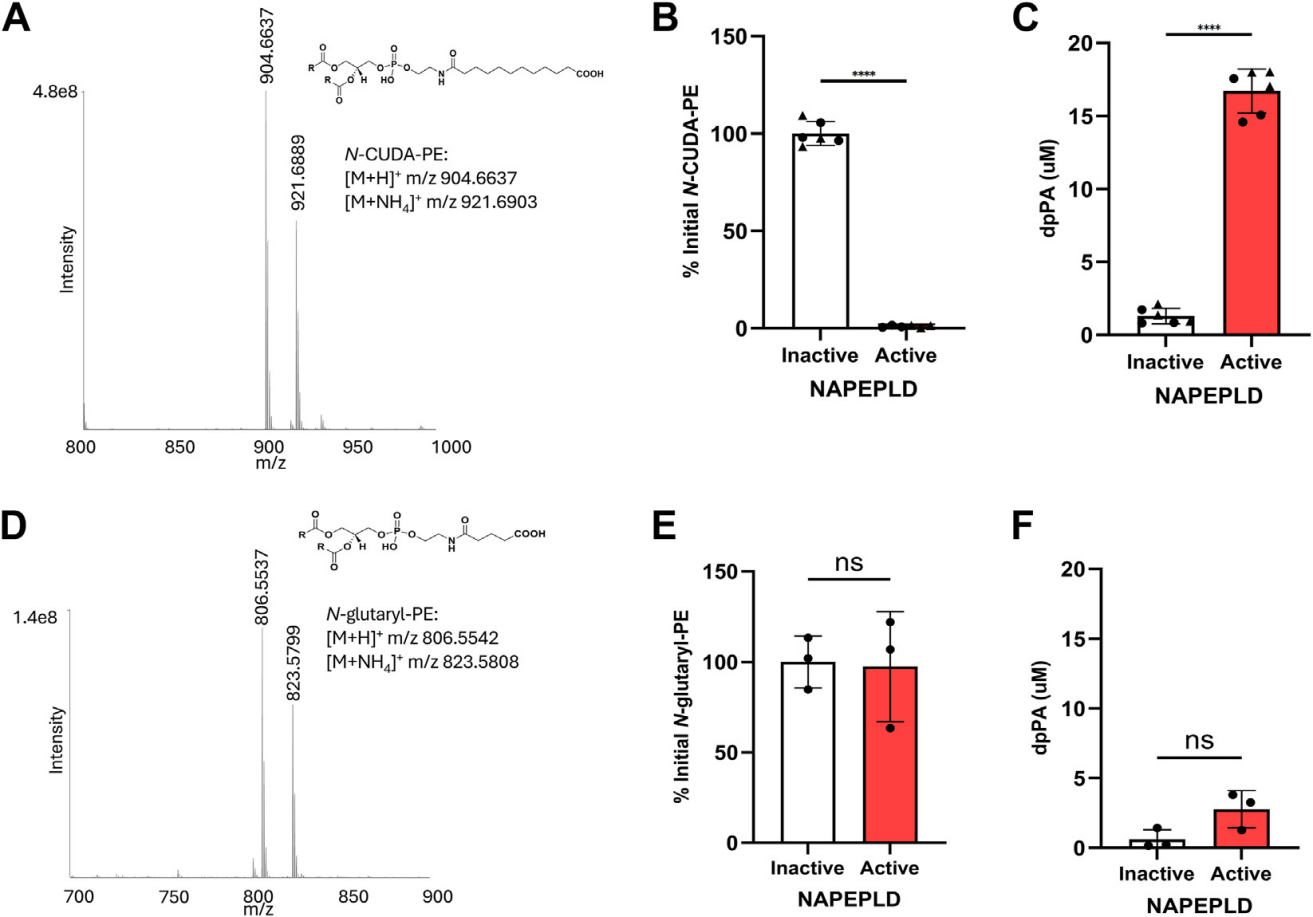
*N*-CUDA-PE and *N*-glutaryl-PE are hydrolyzed by NAPE-PLD. A: *N*-CUDA-PE was purchased from a commercial source and analyzed using Q Exactive Orbitrap mass spectrometer in positive ion scanning mode. The putative structure of *N*-CUDA-PE is shown. Active or heat-inactivated NAPE-PLD was incubated with *N*-CUDA-PE for 2 h, and changes in levels of *N*-CUDA-PE (B) and dipalmitoyl-PA (dpPA) (C) were determined by LC/MS. D: *N*-glutaryl-PE were purchased from a commercial source and analyzed by LC/MS in a similar manner as for *N*-CUDA-PE. Active or heat-inactivated NAPE-PLD was incubated with *N*-glutaryl-PE for 2 h, and changes in levels of *N*-glutaryl-PE (E) and dpPA (F) were determined by LC/MS. For each, mean ± SEM is shown with Student’s *t*-test comparison of active versus inactive enzyme, ns, nonsignificant, ∗∗∗*P* < 0.001, and ∗∗∗∗*P* < 0.0001.

To further investigate the effect of the terminal carboxylate, we purchased *N*-CUDA-PE and *N*-glutaryl-PE and confirmed these products by LC/MS (Fig. 9A, D and supplemental Fig. S14A, D). *N*-CUDA-PE differs from the *N*-KODA-PE KA species only by the absence of the ketone group at carbon 4. Product ion scanning of the precursor ion for *N*-CUDA-PE generated the expected product ion fragmentation at the phosphodiester bonds (*m/z* 551.5035 and *m/z* 256.1905) but also from additional fragmentation of the ω-terminal carboxylate of the headgroup (*m/z* 210.1852) (supplemental Fig. S15), similar to what was seen with both *N*-KODA-PE adducts. Incubation of *N*-CUDA-PE with NAPE-PLD for 2 h resulted in its complete hydrolysis (Fig. 9B and supplemental Fig. S14B) and robust increases in PA formation (15.06 μM) (Fig. 9C and supplemental Fig. S14C). We were also able to detect robust signal for the *N*-CUDA-ethanolamine product (supplemental Fig. S7E). In contrast, incubation of *N*-glutaryl-PE with NAPE-PLD resulted in no hydrolysis (Fig. 9E and supplemental Fig. S14E) or significant increases in PA formation (Fig. 9F and supplemental Fig. S14F).

### Comparing hydrolysis rate of NAPE-PLD substrates

**Fig. 10:**
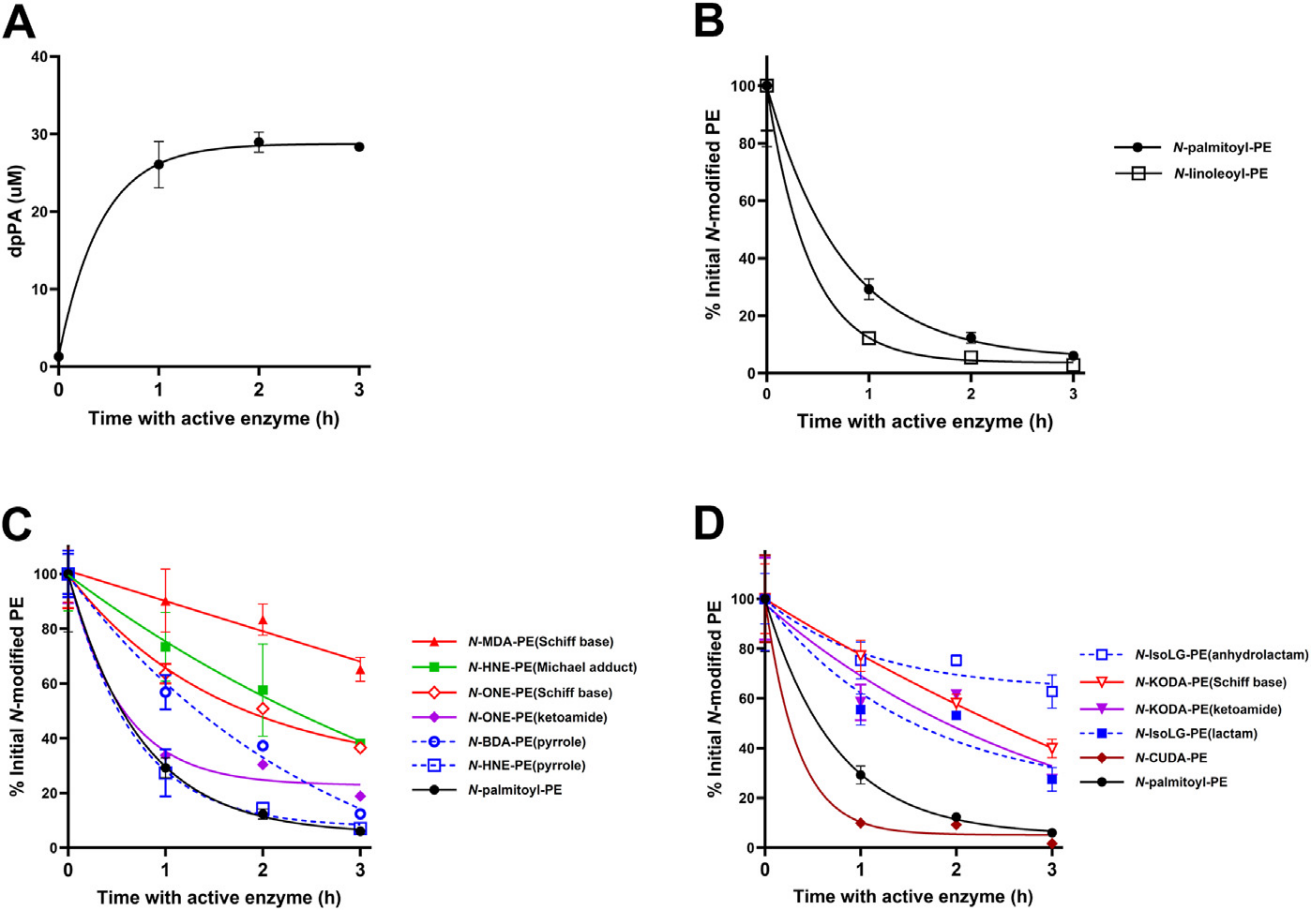
Individual species of NALPEs significantly vary in their relative rate of hydrolysis by NAPE-PLD. Active NAPE-PLD was incubated with a substrate mixture containing approximately equimolar amounts of *N*-palmitoyl-PE, *N*-linoleoyl-PE, *N*-MDA-PE, *N*-BDA-PE, *N*-HNE-PE, *N*-ONE-PE, *N-*KODA-PE, *N-*CUDA-PE, and *N*-IsoLG-PE, and aliquots were removed after 1, 2, and 3 h of incubation and analyzed by LC/MS. Levels of each starting lipid were normalized to values obtained when the substrate mixture was incubated with heat-inactivated NAPE-PLD (0 h incubation with active enzyme). A) Dipalmitoyl-PA (dpPA) levels. B–D: For readability, the results are separated for NAPEs (B), short- and medium-chain NALPEs (C), and NALPE species with carboxylate moiety (D) with the same *N*-palmitoyl-PE data shown on each graph for reference.

During oxidative stress, NAPE-PLD is likely to encounter multiple possible substrates at the same time, so that the relative hydrolysis rate for each substrate will likely determine the extent to which each is degraded. To compare the relative rate that various NAPE-PLD substrates are hydrolyzed under competitive conditions, we created a substrate mixture by combining two NAPEs, *N*-palmitoyl-PE and *N*-linoleoyl-PE, with the various synthetic NALPEs, all in approximately equal amounts. We then incubated this substrate mixture with NAPE-PLD and monitored the progress of the reaction by observing the formation of PA (Fig. 10A) and the loss of each starting substrates (Fig. 10B-D). (For ease of visualization, results for the individual substrates within the mixture are separated into three graphs, with the same *N*-palmitoyl-PE curve shown in each for reference.)

The two canonical NAPE-PLD substrates, *N*-palmitoyl-PE and *N*-linoleoyl-PE (Fig. 10B), as well as *N*-HNE-PE (pyrrole) (Fig. 10C), and *N*-CUDA-PE (Fig. 10D) all exhibited similarly high rates of hydrolysis. *N*-BDA-PE (pyrrole) and *N*-ONE-PE (KA) were hydrolyzed slightly slower (Fig. 10C). *N*-KODA-PE (KA) (Fig. 10C) and *N*-IsoLG-PE (Ltm) (Fig. 10D) were hydrolyzed at a similar rate that was slightly slower than *N*-ONE-PE (KA). *N*-ONE-PE (SB) was hydrolyzed significantly slower than *N*-ONE-PE (KA) (Fig. 10C), and *N*-KODA-PE (SB) was hydrolyzed somewhat slower than *N*-KODA-PE (KA) (Fig. 10D). *N*-HNE-PE (Michael adduct) was hydrolyzed at a similar rate to *N-*ONE-PE (SB) (Fig. 10C). *N*-IsoLG-PE (AL) and *N*-MDA-PE (SB) were hydrolyzed at the slowest rates (Fig. 10C, D).

## Discussion

Previous research has documented the formation of NALPE species both in vitro and in vivo (8, 9, 11, 12, 32, 33, 34), but little has been known about how these molecules are metabolized. In the studies reported here, we demonstrate that many NALPEs are robust substrates for NAPE-PLD, thereby expanding the potential role of this enzyme to include regulating the levels of these bioactive lipids. Our present study also extended the total number of known NALPE species from previous studies that showed the formation of *N*-IsoLG-PE, *N*-MDA-PE, *N*-ONE-PE, *N*-HNE-PE, and *N*-hexanoyl-PE (8, 9, 32, 33, 34, 35, 36, 37, 38, 39). For instance, we identified the formation of *N*-azeoyl-PE, *N*-HODA-PE, and *N*-KODA-PE, the latter of which is consistent which previous studies, which characterized lysine and DNA adducts of KODA (40).

Our interest in identifying enzymes that metabolize various NALPEs arose from previous studies reporting significant biological activity for several NALPEs. For instance, *N*-HNE-PE and *N*-ONE-PE affect the function and activity of membrane proteins such as UCP1 by changing membrane fluidity and are suggested to affect other membrane proteins by a similar mechanism (41). NALPEs such as *N*-MDA-PE, *N*-HNE-PE, *N*-ONE-PE, and *N*-IsoLG-PE induce the proinflammatory responses of endothelial cells (11). *N*-IsoLG-PEs also induce endoplasmic reticulum stress in endothelial cells (42). *N*-IsoLG-PE also strongly activated NF-κB and induced inflammatory cytokine expression in macrophages via receptor for advanced glyation end products (11, 43). We had previously found that NAPE-PLD could hydrolyze *N*-IsoLG-PE to reduce its proinflammatory effects (22), and therefore hypothesized that NAPE-PLD might also hydrolyze other NALPEs.

Our studies extend the structure-activity relationships for NAPE-PLD well beyond that previously established for NAPEs (16, 44). For instance, the presence of an ω-carboxylate group on the N-acyl chain appears to have little detrimental effect on NAPE-PLD hydrolysis when the acyl chain is long but abolishes activity when the acyl chain is short (e.g., *N*-CUDA-PE is hydrolyzed as rapidly as either of the two tested NAPEs, but *N*-glutaryl-PE is not hydrolyzed). One possible explanation for this would be if an extended acyl chain positions the polar ω-carboxylate group sufficiently far away from a hydrophobic channel in the active site that it only minimally disrupts hydrophobic interactions between the acyl carbons proximal to the amide bond and the hydrophobic channel of the enzyme. This notion is consistent with the finding that NALPEs with a ketone group, three carbons from the amide bond (i.e., the KA species) were somewhat less favorable substrates than NAPEs (e.g., the slower rate of hydrolysis for *N*-ONE-PE (KA) than either NAPE or the slower rate of hydrolysis for the *N*-KODA-PE (KA) compared with *N*-CUDA-PE, which only differ by the ketone on carbon 4. Another important structure-activity relationship insight this study provides is the effect of alternative bonds linking the lipid chain to the PE nitrogen. NALPEs with *N*-pyrrole linkages were rapidly hydrolyzed, with the addition of an alkyl chain to the pyrrole, further increasing the rate of hydrolysis (e.g., *N*-HNE-PE [pyrrole] vs *N*-BDA-PE [pyrrole]). In contrast, the SB species of NALPEs such as *N*-MDA-PE, *N*-ONE-PE, and *N*-KODA-PE were poorly hydrolyzed when presented within a mixture that included other substrates. Finally, NALPEs that had two alkyl tails extending from the nitrogen linkage, such as the *N*-HNE-PE (Michael adduct) species and the various *N*-IsoLG-PE species, were hydrolyzed more slowly than those with a single tail in the competition experiments, suggesting that NAPE-PLD is somewhat less able to accommodate such substrates. Altogether, our finding that NAPE-PLD can robustly hydrolyze a wide variety of NALPEs raise the possibility that NAPE-PLD might play an important role in regulating their levels in vivo and that some NALPEs may be more likely to accumulate in vivo due to slower degradation.

Inflammatory conditions, including atherosclerosis, ulcerative colitis, and neurodegenerative diseases, show reduced *NAPEPLD* expression (45, 46, 47). The finding that hepatocyte-specific deletion of *Napepld* renders mice more susceptible to inflammation (48) and adipocyte-specific deletion results in low-grade inflammation (49) suggests that the loss of NAPE-PLD has a causative role in inflammation. Furthermore, bone marrow-derived macrophages lacking *Napepld* have reduced capacity to carry out efferocytosis (50), a critical step in the resolution of inflammation. Our finding that NAPE-PLD robustly hydrolyzes proinflammatory NALPEs raises the possibility that some of the proinflammatory effects of reduced *Napepld* expression could arise from increases in the levels of NALPEs. Future studies are needed to assess whether loss of *Napepld* increases NALPE levels in various tissues and whether this contributes to the proinflammatory phenotype associated with loss of *Napepld*.

## Data availability

All data supporting the findings of this study are available within this article and its supplemental data.

## Supplemental data

This article contains supplemental data.

## Conflict of interest

The authors declare that they have no conflicts of interest with the contents of this article.
