# Supplementary material for: N-aldehyde-modified phosphatidylethanolamines generated by lipid peroxidation are robust substrates of N-acyl phosphatidylethanolamine phospholipase D
Source: J Lipid Res. 2025 May 21;66(7):100831. doi: 10.1016/j.jlr.2025.100831 (PMC12214272; doi:10.1016/j.jlr.2025.100831)
Supplement: Supporting information [file mmc4.docx]

SUPPLEMENTAL INFORMATION

***N*-Aldehyde-Modified Phosphatidylethanolamines generated by lipid peroxidation are robust substrates of *N*-Acyl Phosphatidylethanolamine Phospholipase D**

Reza Fadaei^1^, Annie C. Bernstein^2^, Andrew N. Jenkins^3^, Allison G. Pickens^4^, Jonah E. Zarrow^1^, Abdul-Musawwir Alli-Oluwafuyi^1^, Keri A. Tallman^5^, and Sean S. Davies^1,6*^

^1^Department of Pharmacology, Vanderbilt University. Nashville, TN, USA, 37232; ^2^College of Arts and Sciences, Vanderbilt University, Nashville, TN, USA; ^3^Department of Cell Biology and Physiology, and ^4^Department of Plant and Wildlife Sciences, Brigham Young University. Provo, UT, 84602; ^5^Department of Chemistry, Vanderbilt University, Nashville, TN, USA; and ^6^Vanderbilt Institute of Chemical Biology, Vanderbilt University, Nashville, TN, USA, 37235.

*To whom correspondence should be addressed.


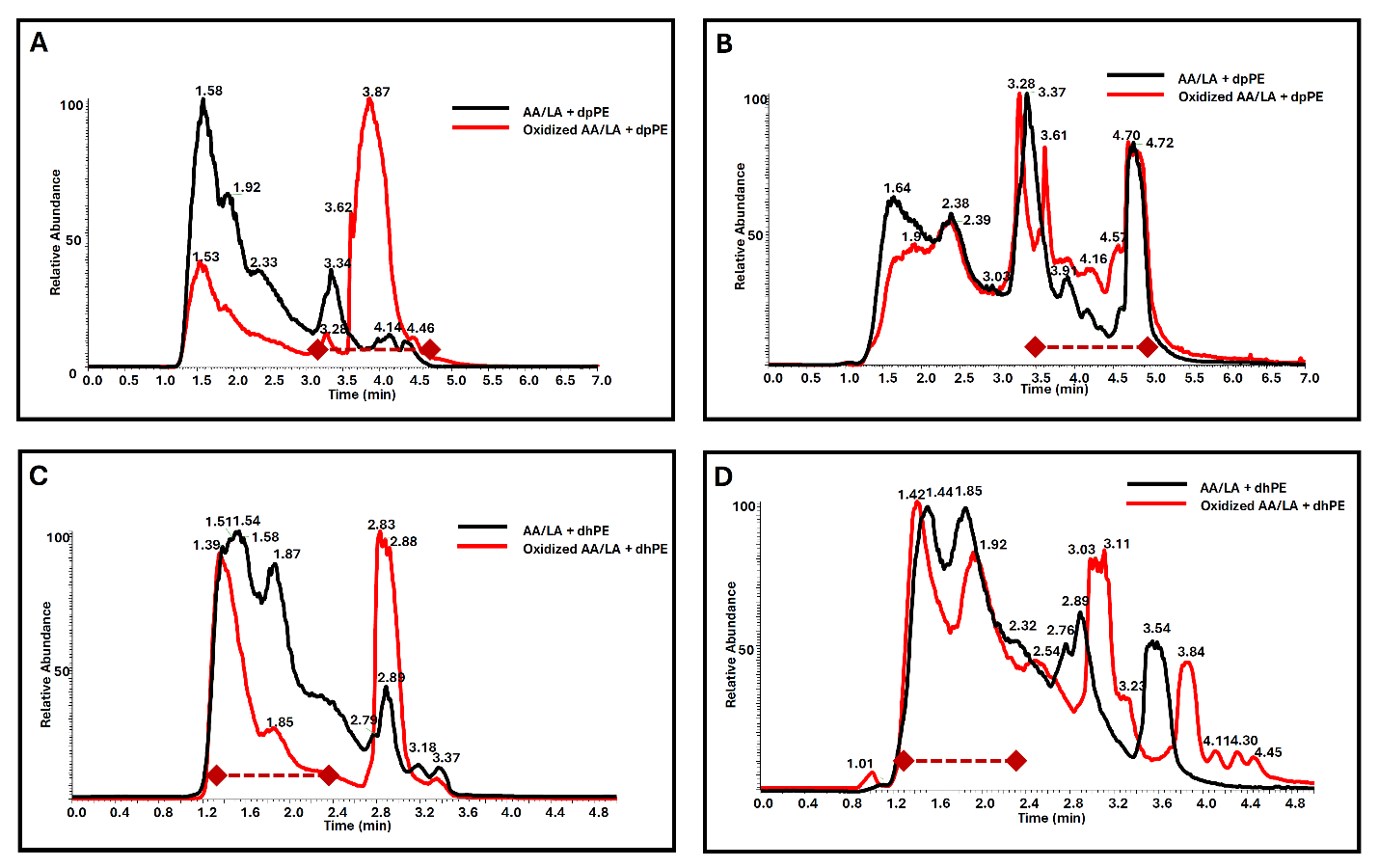


**Supplemental Figure 1. Chromatographs from high resolution mass scanning of PE incubated with linoleic acid and arachidonic acid in the presence and absence of oxidizer (V70).** Arachidonic acid and linoleic acid were oxidized in the presence of dipalmitoyl-PE (dpPE) or dihexanoyl-PE (dhPE) by addition of V70 as a radical initiator. The resulting products were analyzed using an Exactive Orbitrap mass spectrometer in both positive and negative ion scanning modes so that a total of eight analysis were performed. For each chromatograph above, the red trace represents the sample where the oxidizer was added and the black trace represents the sample where no oxidizer was added with the retention time of the oxidized samples used for spectrums shown in Figure 2 indicated by (♦―♦).A, dpPE samples analyzed in negative ion mode; B, dpPE samples analyzed in positive ion mode; C, dhPE samples analyzed in negative ion mode; D, dhPE samples analyzed in positive ion mode.


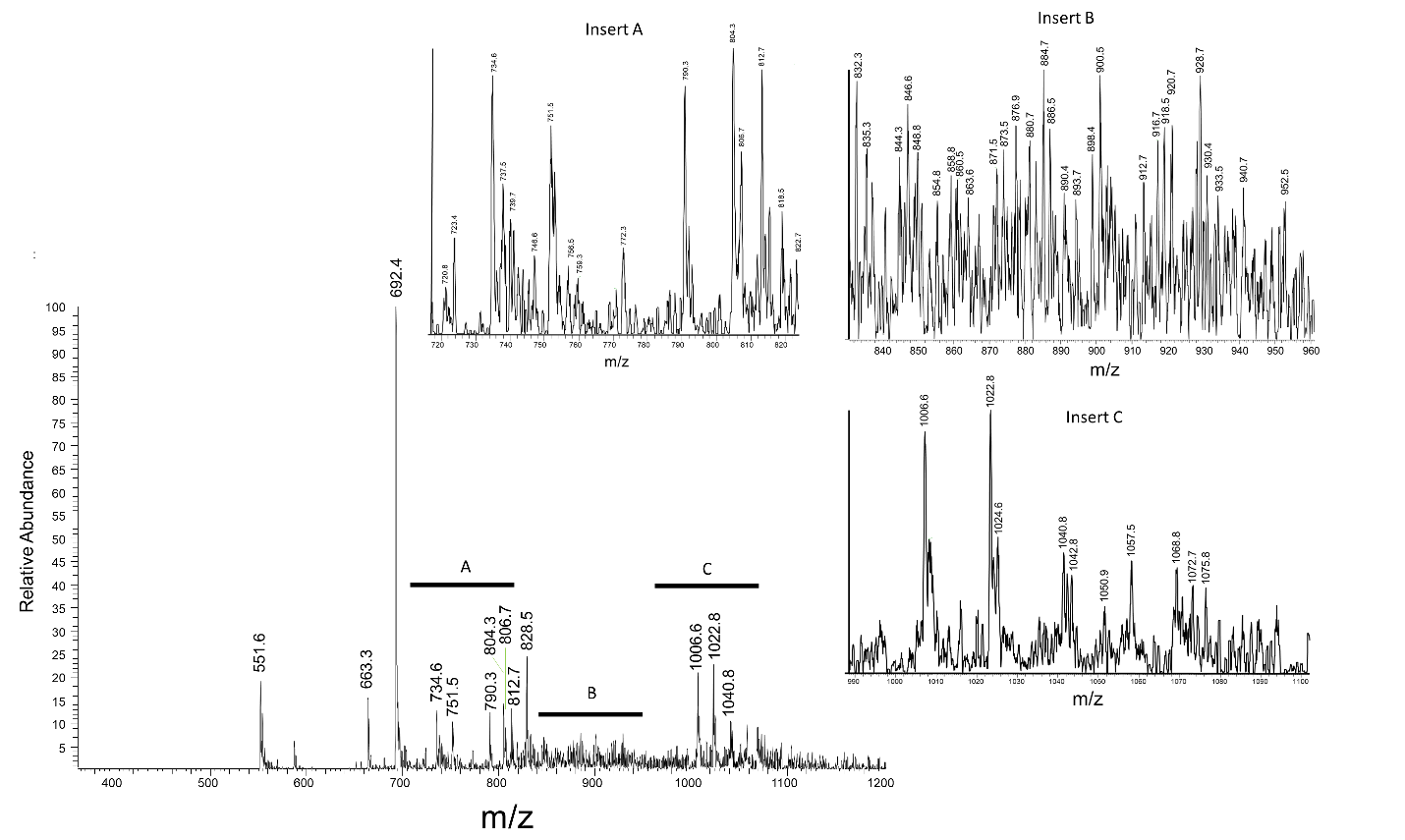


**Supplemental Figure 2. Identification of major NALPE species formed during lipid peroxidation in the presence of dipalmitoyl PE (dpPE) by precursor scanning (triple quadrupole MS).** Arachidonic acid and linoleic acid were oxidized in the presence of dhPE and thhe resulting products were analyzed by LC/MS in positive ion mode using precursor scanning with *m/z* 551.5 as product ion. The mass spectrum of the major broad chromatographic peak is shown with magnified parts indicated by insert A, B and C. The full list of NALPEs detected by this analysis (peak height >3% peak height of PE) is provided in Supplemental Table 2.


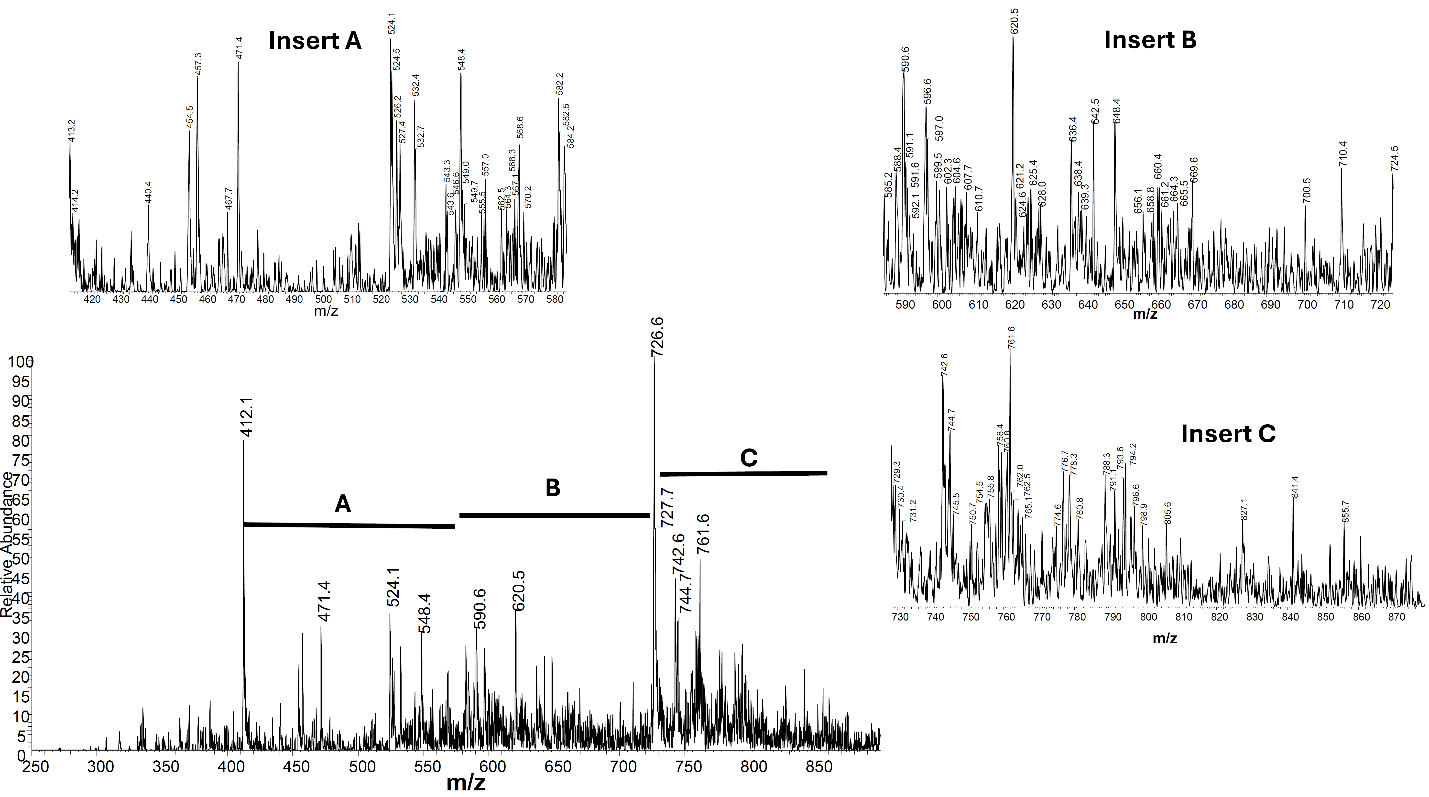


**Supplemental Figure 3. Identification of major NALPE species formed during lipid peroxidation in the presence of dihexanoyl PE (dhPE) by precursor scanning (triple quadrupole MS).** Arachidonic acid and linoleic acid were oxidized in the presence of dhPE and the resulting products were analyzed by LC/MS in positive ion mode using precursor scanning with *m/z* 271.2 as product ion. The mass spectrum of the major broad chromatographic peak is shown with magnified parts indicated by insert A, B and C. The full list of NALPEs detected by this analysis (peak height >3% peak height of PE) is provided in Supplemental Table 2.

**
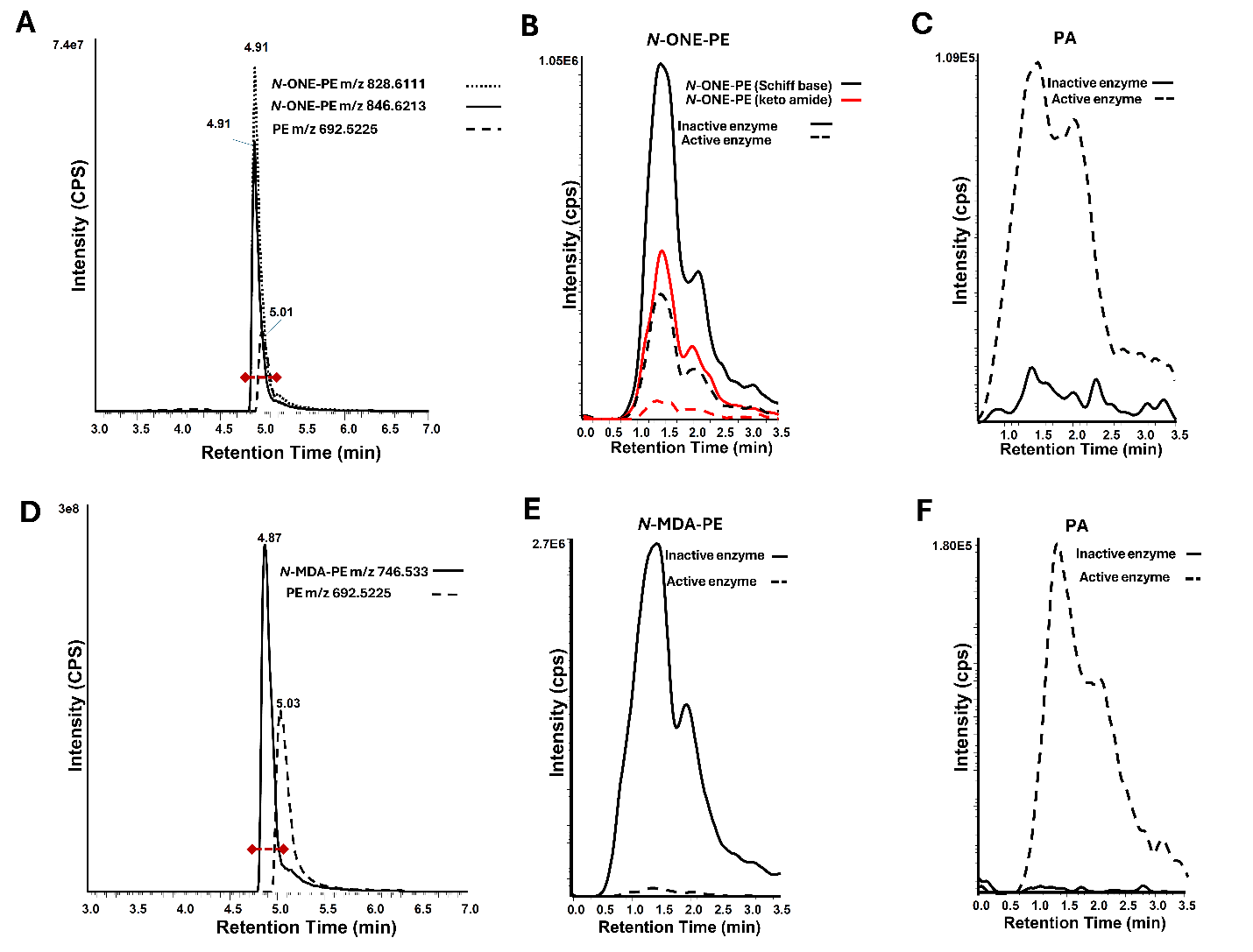
Supplemental Figure 4. Chromatograms corresponding to triple quadrupole LC/MS spectra and NAPEPLD hydrolysis of *N*-ONE-PE and *N*-MDA-PE in Figure 6.** A) Precursor scanning chromatogram (product ion *m/z 551.5*) of the reaction of ONE with PE, showing the formation of *N*-ONE-PE, including Schiff base and ketoamide species. Solid line represents the total ion current from precursor scan with retention time used for spectra shown in Figure 6A indicated by (♦―♦). Dotted lines represent reconstructed single ion current for major ions found in the spectrum showing *N*-ONE-PE species. B) LC/MS in MRM mode to monitor *N*-ONE-PE species and C) phosphatidic acid (PA) as product of the reaction. D) Precursor scanning chromatogram (product ion *m/z 551.5*) of the reaction of MDA with PE, resulting in the formation of N-MDA-PE Schiff base (aka *N*-propenal-PE). Solid line represents the total ion current of the precursor scan with retention time used for the spectra shown in Figure 6D indicated by (♦―♦). Dotted lines represent reconstructed single ion current for major ions found in the spectrum showing *N*-MDA-PE(Schiff base). E) LC/MS in MRM mode to monitor *N*-MDA-PE(Schiff base) and F) phosphatidic acid (PA) as product of the reaction.


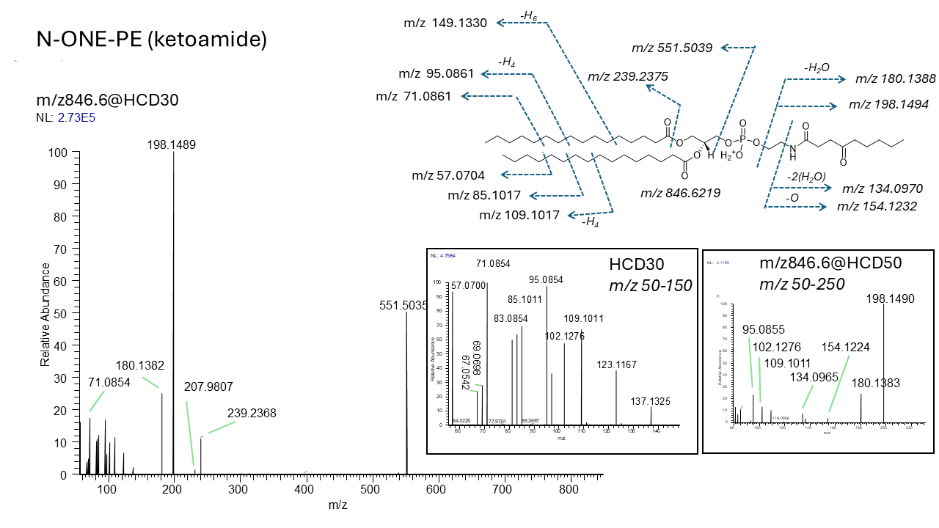


**Supplemental Figure 5. Product ion scans of putative *N*-ONE-PE ketoamide by high resolution mass spectrometry**. Product scanning for precursor ion m/z 846.6 using High-energy collisional dissociation (HCD) with energies set from 10 to 50 were performed on the Q Exactive instrument to generate high resolution product scans. Shown here is the full spectrum for the HCD30 scan and the interpretation of the fragmentation results. The insert on the left shows a zoomed in limited scan spectrum from the same analysis to provide more detail of the ions with lower m/z that represent fragmentation of the PE moiety. The right insert provides the limited mass scan spectrum of the HCD50 analysis.

**
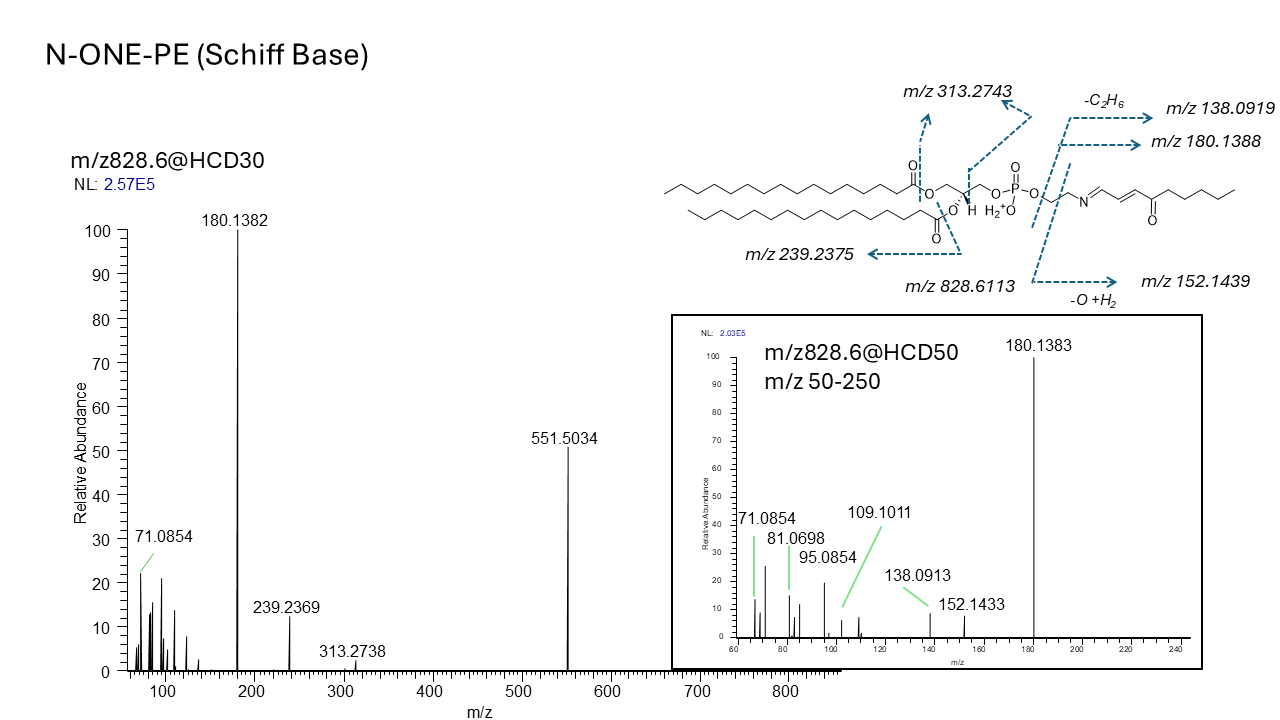
Supplemental Figure 6. Product ion scans of putative *N*-ONE-PE Michael Adduct by high resolution mass spectrometry.** Product scanning for precursor ion m/z 828.6 using High-energy collisional dissociation (HCD) with energies set from 10 to 50 were performed on the Q Exactive instrument to generate high resolution product scans. Shown here is the full spectrum for the HCD30 scan and the interpretation of the fragmentation results. The insert shows a zoomed in limited scan spectrum from the limited mass scan spectrum of the HCD50 analysis to show some of the ions unique to this precursor.

**
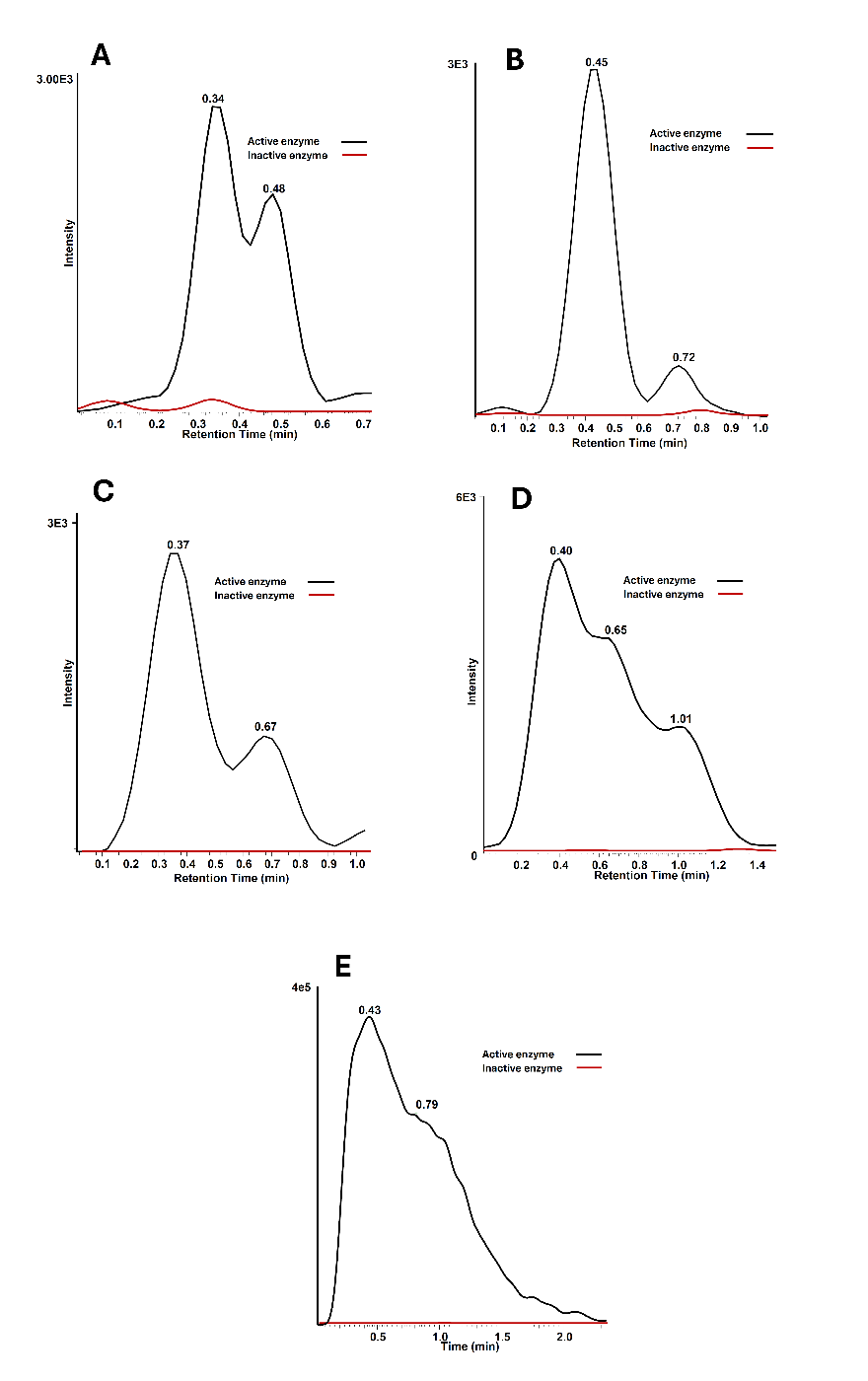
**

**Supplemental Figure 7. Chromatographs from multiple reaction monitoring for the N-aldehyde modified-ethanolamines produced by incubation of individual NALPEs with NAPE-PLD.** Multiple reaction monitoring for the appropriate *N*-aldehyde modified ethanolamine (NALEs) was performed using the precursor and product ion values given in Table 1 in the same experiment shown in Figures 6 to 9. Shown here are the resulting MRM chromatographs for the individual NALEs generated in incubations with inactive (red trace) or active NAPE-PLD (black trace) where signal was detected for these MRM chromatographs. A. *N*-ONE-ethanolamine ketoamide. B. *N*-IsoLG-ethanolamine. C. *N*-KODA-ethanolamine Schiff base. D. *N*-KODA-ethanolamine ketoamide. E. *N*-CUDA-ethanolamine.

**
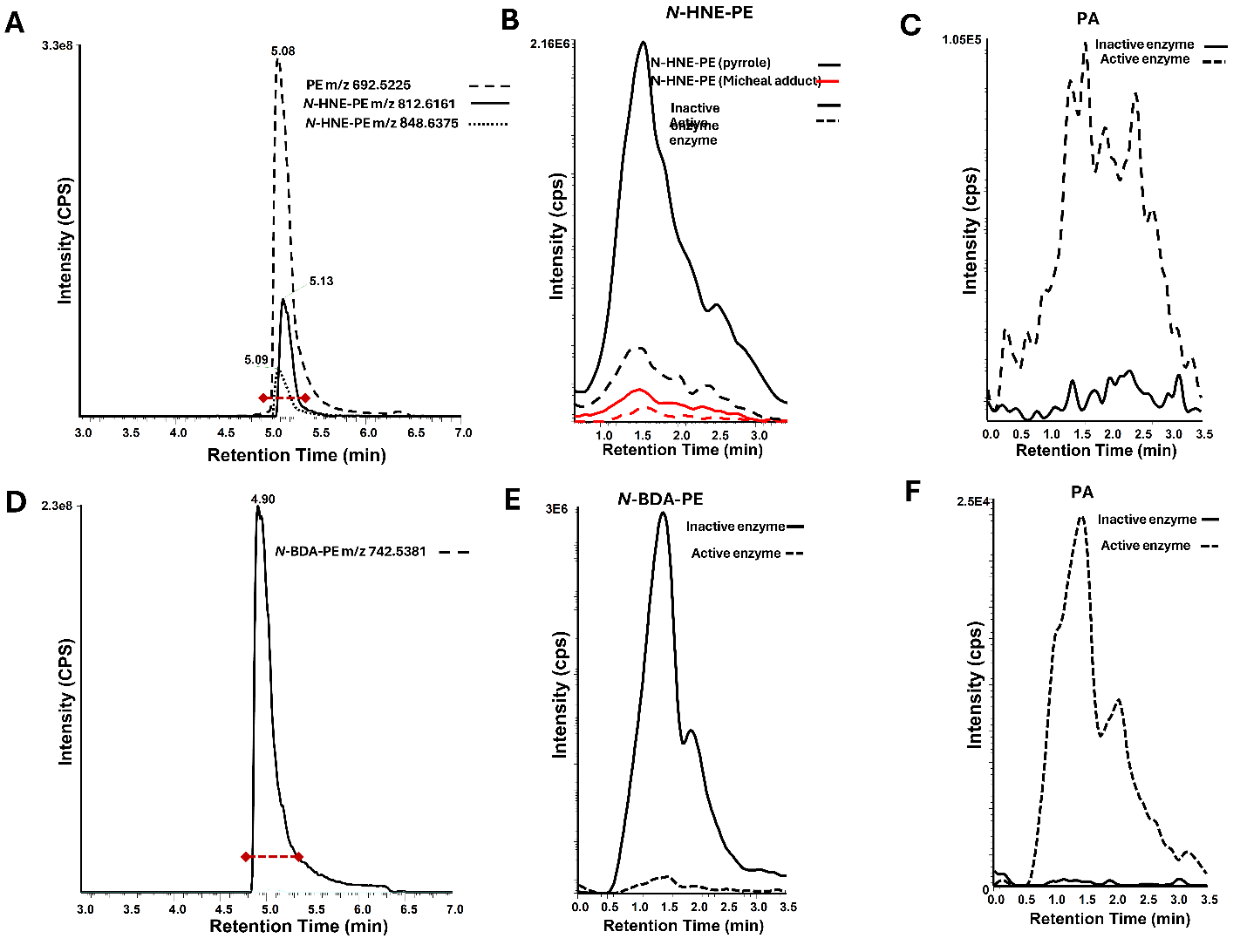
Supplemental Figure 8. Chromatograms corresponding to LCMS spectra NAPEPLD hydrolysis of *N*-HNE-PE and *N*-BDA-PE in Figure 7.** A) Precursor scanning chromatogram (product ion *m/z 551.5*) of the reaction of HNE with PE, resulting in the formation of *N*-HNE-PE, including pyrrole and Michael adduct species. Solid line represents the total ion current from precursor scan with retention time used for spectra shown in Figure 7A indicated by (♦―♦). Dotted lines represent reconstructed single ion current for major ions found in the spectrum showing *N*-HNE-PE spices. B) LC/MS in MRM mode to monitor *N*-HNE-PE species and C) phosphatidic acid (PA) as product of the reaction. D) Precursor scanning chromatogram (product ion *m/z 551.5*) of the reaction of BDA with PE, resulting in the formation of *N*-BDA-PE(pyrrole) (aka *N*-pyrrole-PE). Solid line represents the total ion current from precursor scan with retention time used for spectra shown in Figure 7D indicated by (♦―♦). Dotted lines represent reconstructed single ion current for major ions found in the spectrum showing *N*-pyrrole-PE. E) LC/MS in MRM mode to monitor *N*-pyrrole-PE and F) phosphatidic acid (PA) as product of the reaction.


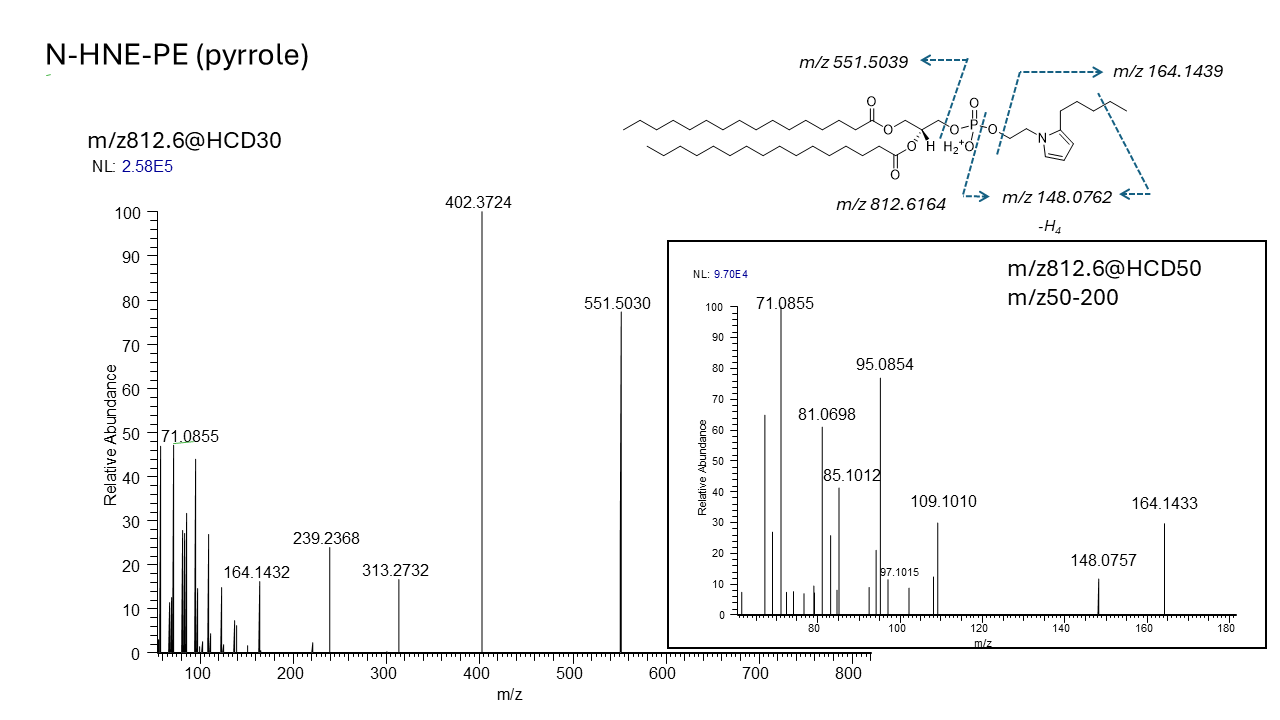


**Supplemental Figure 9. Product ion scans of putative *N*-HNE-PE pyrrole by high resolution mass spectrometry.** Product scanning for precursor ion m/z *812.6* using High-energy collisional dissociation (HCD) with energies set from 10 to 50 were performed on the Q Exactive instrument to generate high resolution product scans. Shown here is the full spectrum for the HCD30 scan and the interpretation of the fragmentation results. The insert shows a zoomed in limited scan spectrum from the limited mass scan spectrum of the HCD50 analysis to show some of the products ions unique to this precursor.

**
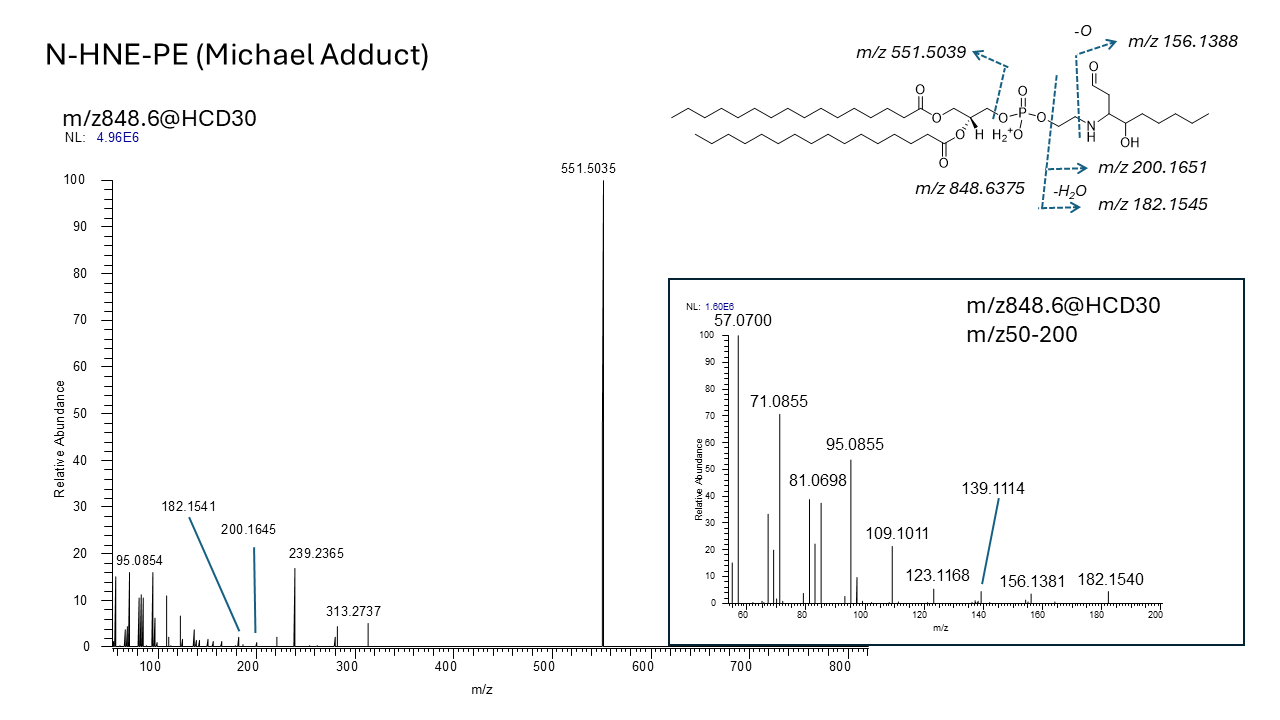
Supplemental Figure 10. Product ion scans of putative *N*-HNE-PE Michael Adduct by high resolution mass spectrometry.** Product scanning for precursor ion m/z *848.6* using High-energy collisional dissociation (HCD) with energies set from 10 to 50 were performed on the Q Exactive instrument to generate high resolution product scans. Shown here is the full spectrum for the HCD30 scan and the interpretation of the fragmentation results. The insert shows a zoomed in limited scan spectrum from the limited mass scan spectrum of the HCD50 analysis to show some of the products ions unique to this precursor.

**
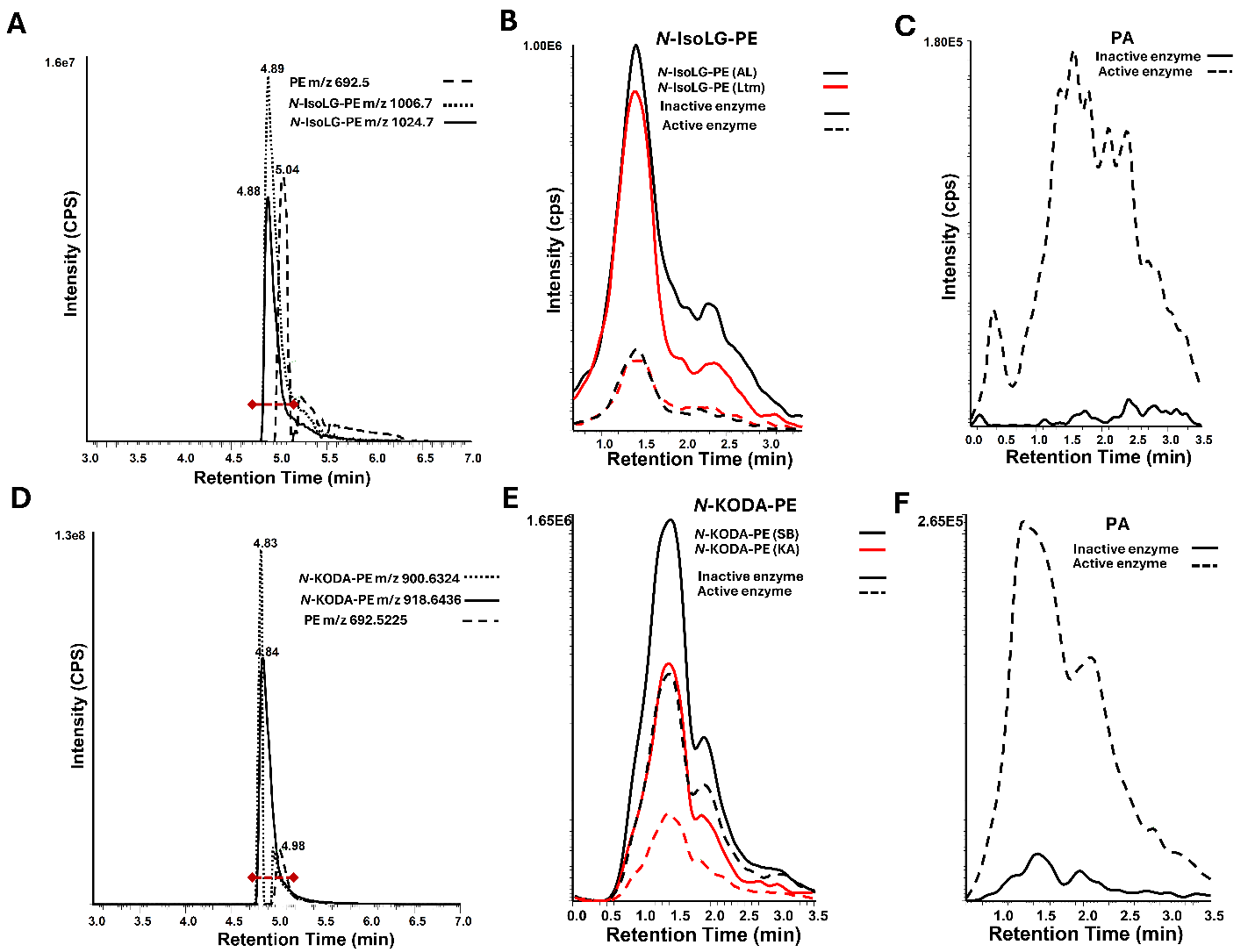
Supplemental Figure 11. Chromatograms corresponding to LCMS spectra and NAPEPLD hydrolysis of *N*-IsoLG-PE and *N*-KODA-PE in Figure 8.** A) Precursor scanning chromatogram (product ion *m/z 551.5*) of the products of the reaction of IsoLG with PE, resulting in the formation of *N*-IsoLG-PE, including anhydrolactam and lactam species. Solid line represents the total ion current from precursor scan with retention time used for spectra shown in Figure 8A indicated by (♦―♦). Dotted lines represent reconstructed single ion current for major ions found in the spectrum showing *N*-IsoLG-PE spices. B) LC/MS in MRM mode to monitor *N*-IsoLG-PE species and C) phosphatidic acid (PA) as product of the reaction. D) Precursor scanning chromatogram (product ion *m/z 551.5*) of the products of the reaction of KODA with PE, resulting in the formation of *N*-KODA-PE species including Schiff base and ketoamide. Solid line represents the total ion current from precursor scan with retention time used for spectra shown in Figure 8D indicated by (♦―♦). Dotted lines represent reconstructed single ion current for major ions found in the spectrum showing *N*-KODA-PE spices. E) LC/MS in MRM mode to monitor *N*-KODA-PE species and F) phosphatidic acid (PA) as product of the reaction.

**
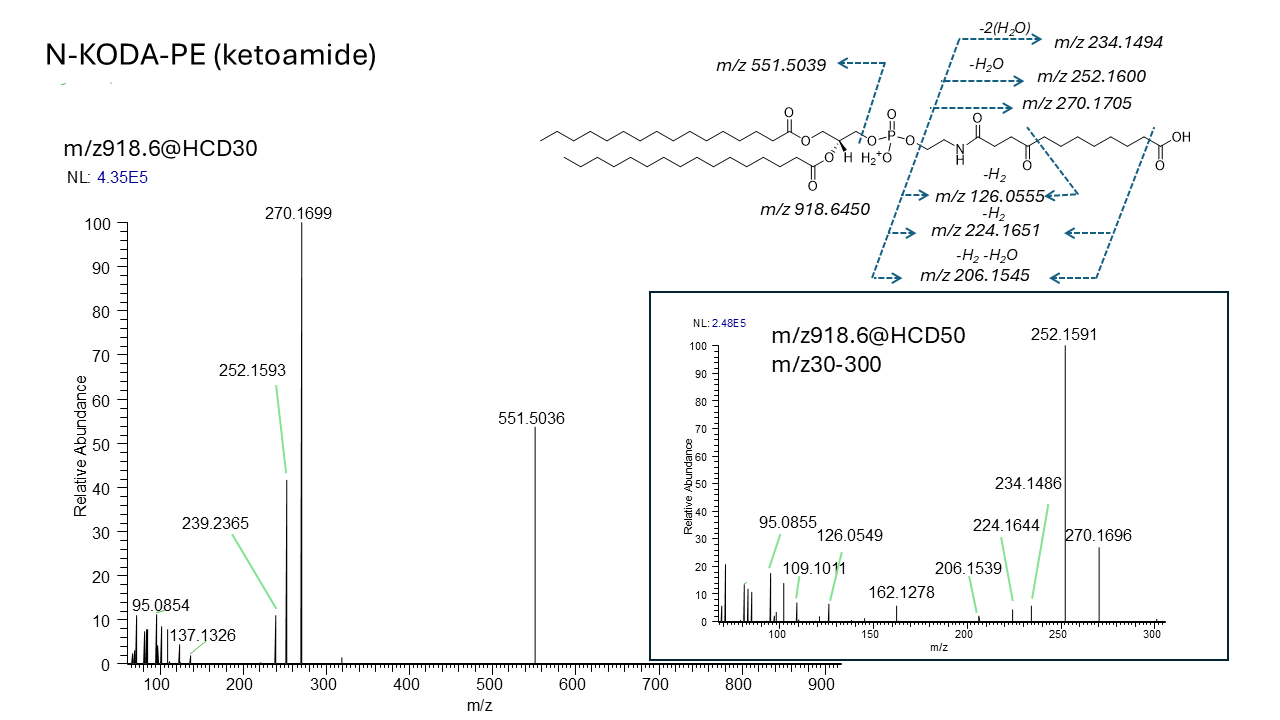
Supplemental Figure 12. Product ion scans of putative *N*-KODA-PE ketoamide by high resolution mass spectrometry.** Product scanning for precursor ion m/z *918.6* using High-energy collisional dissociation (HCD) with energies set from 10 to 50 were performed on the Q Exactive instrument to generate high resolution product scans. Shown here is the full spectrum for the HCD30 scan and the interpretation of the fragmentation results. The insert shows a zoomed in limited scan spectrum from the limited mass scan spectrum of the HCD50 analysis to show some of the products ions unique to this precursor.

**
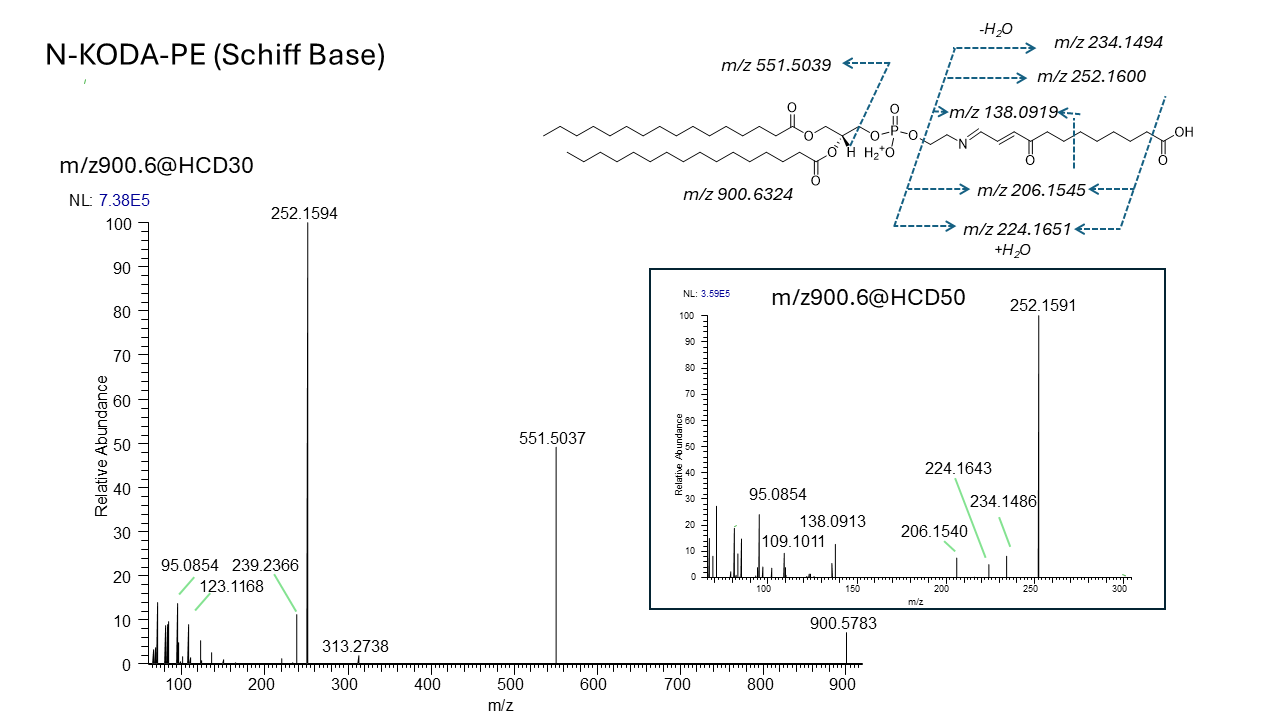
Supplemental Figure 13. Product ion scans of putative *N*-KODA-PE Schiff Base by high resolution mass spectrometry.** Product scanning for precursor ion m/z *900.6* using High-energy collisional dissociation (HCD) with energies set from 10 to 50 were performed on the Q Exactive instrument to generate high resolution product scans. Shown here is the full spectrum for the HCD30 scan and the interpretation of the fragmentation results. The insert shows a zoomed in limited scan spectrum from the limited mass scan spectrum of the HCD50 analysis to show some of the products ions unique to this precursor.

**
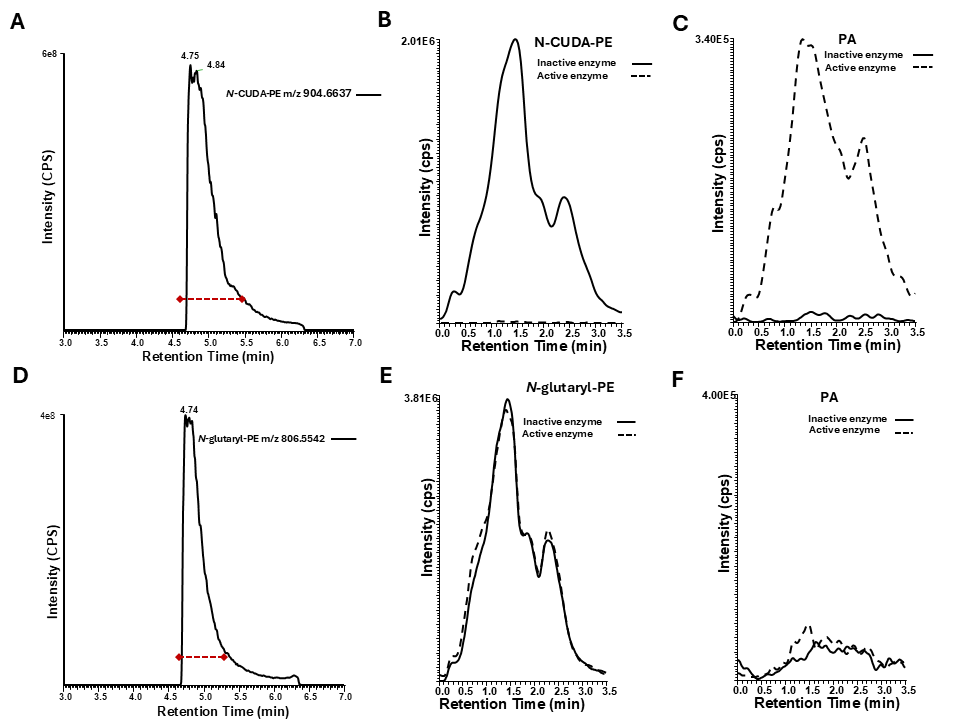
Supplemental Figure 14. Chromatograms corresponding to LCMS spectra and NAPEPLD hydrolysis of *N*-CUDA-PE and *N*-glutaryl-PE in Figure 9.** A) Precursor scanning chromatogram (product ion *m/z 551.5*) of *N*-CUDA-PE commercial preparation. Solid line represents the total ion current from precursor scan with retention time used for spectra shown in Figure 9A indicated by (♦―♦). Dotted lines represent reconstructed single ion current for major ions found in the spectrum showing *N*-CUDA-PE species. B) LC/MS in MRM mode to monitor *N*-CUDA-PE and C) phosphatidic acid (PA) as product of the reaction. D) Precursor scanning chromatogram of commercially available *N*-glutaryl-PE. Solid line represents the total ion current from precursor scan with retention time used for spectra shown in Figure 9D indicated by (♦―♦). Dotted lines represent reconstructed single ion current for major ions found in the spectrum showing *N*-glutaryl-PE. E) LC/MS in MRM mode to monitor *N*-glutaryl-PE and F) phosphatidic acid (PA) as product of the reaction.

**
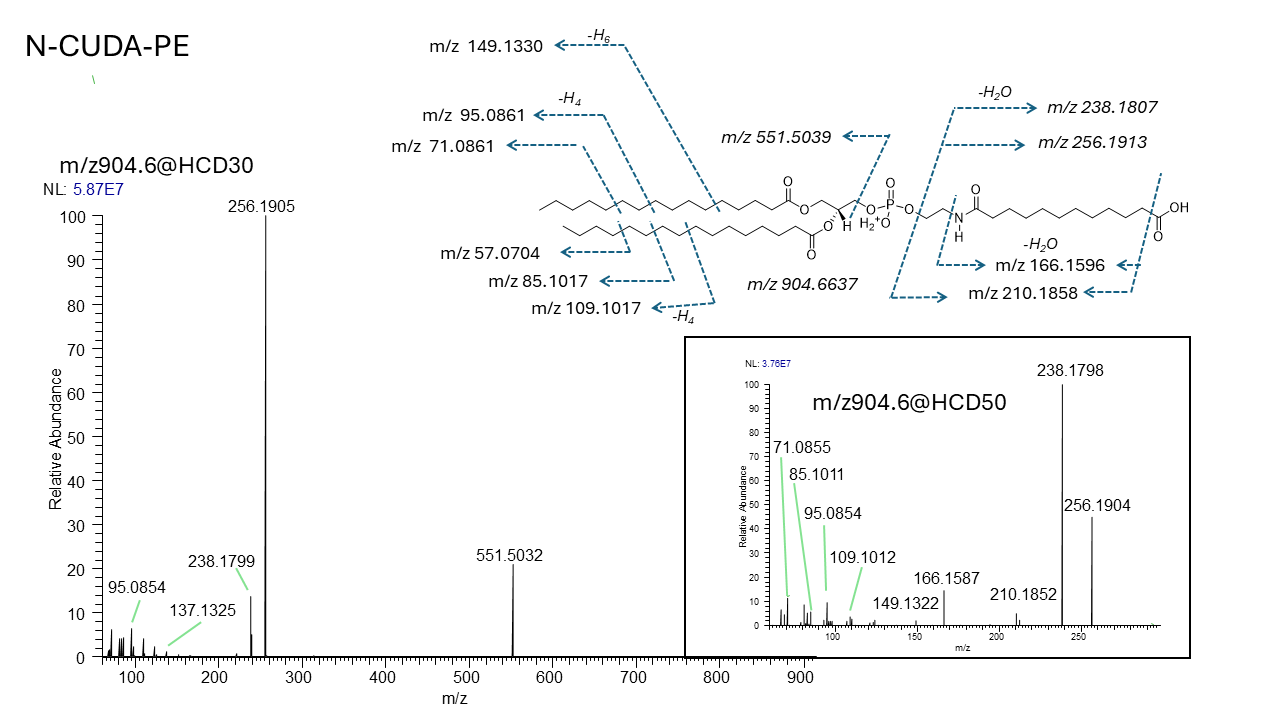
Supplemental Figure 15. Product ion scans of commercial product *N*-CUDA-PE by high resolution mass spectrometry.** Product scanning for precursor ion m/z *904.6* using High-energy collisional dissociation (HCD) with energies set from 10 to 50 were performed on the Q Exactive instrument to generate high resolution product scans. Shown here is the full spectrum for the HCD30 scan and the interpretation of the fragmentation results. The insert shows a zoomed in limited scan spectrum from the limited mass scan spectrum of the HCD50 analysis to show some of the products ions unique to this precursor.
